# APOBEC3G Regulation of the Evolutionary Race Between Adaptive Immunity and Viral Immune Escape Is Deeply Imprinted in the HIV Genome

**DOI:** 10.3389/fimmu.2018.03032

**Published:** 2019-01-11

**Authors:** Faezeh Borzooee, Krista D. Joris, Michael D. Grant, Mani Larijani

**Affiliations:** Immunology and Infectious Diseases Program, Division of Biomedical Sciences, Faculty of Medicine, Memorial University of Newfoundland, St. John's, NL, Canada

**Keywords:** CTL epitope, APOBEC3G (A3G), HIV, immune escape, viral evolution

## Abstract

APOBEC3G (A3G) is a host enzyme that mutates the genomes of retroviruses like HIV. Since A3G is expressed pre-infection, it has classically been considered an agent of innate immunity. We and others previously showed that the impact of A3G-induced mutations on the HIV genome extends to adaptive immunity also, by generating cytotoxic T cell (CTL) escape mutations. Accordingly, HIV genomic sequences encoding CTL epitopes often contain A3G-mutable “hotspot” sequence motifs, presumably to channel A3G action toward CTL escape. Here, we studied the depths and consequences of this apparent viral genome co-evolution with A3G. We identified all potential CTL epitopes in Gag, Pol, Env, and Nef restricted to several HLA class I alleles. We simulated A3G-induced mutations within CTL epitope-encoding sequences, and flanking regions. From the immune recognition perspective, we analyzed how A3G-driven mutations are predicted to impact CTL-epitope generation through modulating proteasomal processing and HLA class I binding. We found that A3G mutations were most often predicted to result in diminishing/abolishing HLA-binding affinity of peptide epitopes. From the viral genome evolution perspective, we evaluated enrichment of A3G hotspots at sequences encoding CTL epitopes and included control sequences in which the HIV genome was randomly shuffled. We found that sequences encoding immunogenic epitopes exhibited a selective enrichment of A3G hotspots, which were strongly biased to translate to non-synonymous amino acid substitutions. When superimposed on the known mutational gradient across the entire length of the HIV genome, we observed a gradient of A3G hotspot enrichment, and an HLA-specific pattern of the potential of A3G hotspots to lead to CTL escape mutations. These data illuminate the depths and extent of the co-evolution of the viral genome to subvert the host mutator A3G.

## Introduction

HIV, like other RNA viruses, evolves rapidly and continuously through the accumulation of mutations (1). The high rate of HIV genome mutation, between 10^−4^ and 10^−5^ mutations per nucleotide per replication cycle, is generated by HIV's error-prone reverse transcriptase (RT) (2–7). APOBEC3G (A3G) is a member of the apolipoprotein B mRNA-editing enzyme catalytic polypeptide-like editing complex (APOBEC) family of cytidine deaminase enzymes. Malim and colleagues, in 2002, discovered that A3G is responsible for the prevalence of G to A mutations in HIV sequences from HIV infected individuals (8). The APOBEC family includes 11 members in humans: activation-induced cytidine deaminase (AID), APOBEC1, APOBEC2, APOBEC3A-H, and APOBEC4, which, through their cytidine deaminase activity, are involved in diverse physiological processes including lipid metabolism, antibody diversification, virus/retroelement restriction, and cancer genome hypermutation (9–13).

In general, the A3 branch family members are capable of impeding infectivity of HIV and several other viral infections such as hepatitis B, human T cell leukemia virus type 1, and human papillomavirus (14–18) though A3G is the most effective actor on the HIV genome (19). These enzymes exert their anti-viral restriction activity by deamination of cytidine to uridine (C to U) in the minus-strand single-stranded DNA during reverse transcription of viral genomic RNA which mediates guanosine to adenosine (G to A) mutation in plus-strand DNA (20–26). A3G is constitutively expressed in resting CD4^+^ T cells, macrophages, and dendritic cells, but can be further induced by interferon (IFN) (27–29). It is packaged into the HIV virion in A3G-expressing producer cells and can act on the viral genome in the subsequently-infected cell (20). It has been shown by single-virion analysis that A3G can be co-packed with A3F, A3D, or A3H haplotype II and co-mutate the same viral genome in a single cycle of HIV replication (30).

A3G has a sequence preference for mutating C in CCC, TCC, and ACC motifs, but this preference is further modulated by the DNA secondary structure, together mediating accumulation of G to A mutations in viral cDNA (18, 21, 22, 24, 30–34). A3G-mediated mutations on the HIV genome also follow a twin gradient pattern, as determined by the central and end polypurine tracts (PPT)'s impact on the reverse transcription dynamics of the HIV genome altering the time that various regions are left single-stranded and available for A3G to act on (34, 35). Depending on the load and positions of A3G-induced mutations, this could lead to either degradation or G to A hypermutation in the viral genome (23, 25, 26). One of the early observations of the contribution of A3G to producing defective viral proteins was its ability to create premature stop codons (36, 37). For example, the codon encoding tryptophan (TGG) is converted by A3G to a stop codon (TGA) (37, 38). A3G is also able to physically interfere with HIV replication in a deamination-independent manner by blocking reverse transcription or binding to tRNA to prevent reverse transcription initiation (39–41). However, it appears that viral restriction has a higher requirement for deamination-dependent A3G activity (42–45).

Over the last decade and a half, several hundred published studies have focused on A3G's HIV restriction activity. A3G can induce as many as five mutations per kilobase (22, 26, 32, 38), at least an order of magnitude higher than RT's error rate. It has been suggested that HIV's RT is only responsible for 2% of HIV genome mutagenesis and the other 98% can be attributed to the action of A3G (46). While high A3G activity correlates with slower disease progression, lower A3G activity leading to sub-lethal mutations might enhance HIV diversity and lead to more rapid disease progression (47). In contrast, other studies showed a much lower contribution of A3G to genetic variation of HIV, as compared with RT-driven mutations. One study reported negligible sub-lethal mutation frequencies as low as 4 × 10^−21^ and 1 × 10^−11^ for A3G and A3F mutations, respectively, which is significantly lower than the frequency of mutations arising from RT (39). Most reports suggest that HIV can experience both a beneficial and a harmful influence from A3G expression (47–50). Other studies reported that A3G is less likely to impose HIV diversification and facilitate viral diversification and adaptation *in vivo*, and that A3G, even at low expression levels, is lethal for HIV (36, 51, 52).

Most studies reporting the impressively high load of A3G mutation were carried out using Vif-deficient HIV because A3G is antagonized by the HIV encoded accessory protein Vif (53–56). Although Vif is necessary for HIV replication in A3G-expressing cells, it is not required in A3G-deficient cells (8, 53–58). Vif binding mediates proteasomal degradation of A3G, but it can also downregulate the translation of A3G (59–61). It has been reported that the accessory protein Vpr can also bind A3G and mediate its proteasomal degradation (62). Thus, in the presence of HIV's full complement of accessory anti-A3G factors, only low levels of mutations are induced by A3G (47, 49, 59, 63, 64).

HIV specific CD8^+^ cytotoxic T cell (CTL) responses and their human leukocyte antigen (HLA) restriction are crucial determinants of viral containment following the initial innate immune response (65, 66). Multiple parameters such as HLA genotype, virus sequence, and T cell receptor repertoire contribute to CTL response effectiveness (67–72). Despite the significant protective role of CTLs in limiting viral HIV replication, the immune system ultimately fails to clear HIV, at least in part because of mutations within or adjacent to CTL epitope during the early and chronic phases of disease progression (73). HIV is under intense evolutionary pressure for escape mutations that lead to evasion of CTL killing and CTL escape mutations are a major force in driving viral evolution in acute/early chronic infection (74, 75). These mutations can be located either inside or outside CTL epitopes, be fast or slow in appearance, but to be selected, they ought to maintain a balance with a cost for viral replication fitness and escape from CTL (76–80). Mutations that facilitate immune evasion are positively selected and become dominant in the viral population (73, 81, 82). On the other hand, sometimes amino acid alterations under immune pressure can even confer a *de novo* immune response (83).

CTL escape mutations can act through several mechanisms: by reducing or abrogating binding of viral epitopes to HLA Class I, disrupting intracellular epitope processing or altering recognition by T cell receptors (84–89). Viral proteins in infected cells are first proteolytically degraded in the cytosol by immunoproteasomes (86, 90, 91). Proteasomal degradation product peptides including epitope precursors can be up to 32 amino acids long; however, immunoproteasomes are inclined to generate longer peptides ending with C-terminal hydrophobic residues that are anchors for most HLA class I molecules. After post-proteasomal degradation, epitope precursors typically 8–16 amino acids in length, are transferred into the endoplasmic reticulum (ER) lumen where HLA class I molecules are folded and assembled, by the transporter-associated-with-antigen-processing (TAP1 and TAP2) (92). Further N-terminal trimming in the ER can occur by enzymes such as the ER aminopeptidases (ERAP1 and 2) to fit the groove of restricting HLA class I molecules (93, 94). The peptide-HLA complex is subsequently transported to the cell surface to be recognized by CTLs. Thus, proteasomal degradation and antigen processing are key determinants of epitope availability for the anti-HIV CTL response (95). In addition to mutations in epitopes, immune escape can also be altered through mutation of the flanking regions that impact proteasomal processing (96). It has been shown that the robustness of the anti-HIV CTL response correlates with the number of epitopes generated due to proteasomal cleavage, and thus, mutations that impact processing can drastically influence CTL escape (77–79, 89, 97–100).

The most restrictive step in antigen processing is the peptide's ability to bind to the specific expressed set of HLA class I molecules using N- and C-terminal anchor residues that bind into the groove of a specific HLA class I molecule (101). Mutations at anchor residues can disrupt HLA class I binding, whereas other mutations such as those at the central bulge of the peptide (normally residues, 4–6 in the canonical 9-mer peptide) interfere with TCR recognition of the HLA-peptide complex (73, 84, 96, 102). It remains to be fully understood how the host's condition shapes the availability of beneficial mutations; however, HLA profile is a major CTL escape driver (85, 103–105). HLA-B is the most protective among all three HLA class I loci (A, B, and C) (106). CTL escape and reversion pathways are more closely associated with epitopes restricted to protective HLA alleles such as HLA-B27 and -B57, which are associated with slower disease progression and more robust HIV-specific CTL responses (78, 107). Escape mutations in these HLA restricted epitopes incur a high fitness cost by reducing viral replicative capacity (108). In contrast, HLA-B35 and -B8 are associated with rapid disease progression based on their ability to present less effective epitopes to TCRs (109–113).

Mutational diversity of HIV's genome has a crucial role in evasion of immune recognition and multiple studies have implicated A3G as an important player in the interplay between the adaptive immune anti-viral CTL response viral adaptation and immune escape (114–119). A3G has been proposed to induce CTL escape in two ways, either by directly mutating CTL epitopes or by causing mutations outside epitopes which influence the peptide degradation and HLA presentation of wild-type CTL epitopes (117, 119–121). Several reports describe A3G-induced mutations located within or flanking CTL epitopes. One report found remarkable evidence for enrichment of non-synonymous amino acid substitutions by A3G in the anchor or proximal amino acid residues of HLA-restricted epitopes that are important in epitope processing leading to immune escape (115). Consistent with a role for A3G in CTL escape, an earlier bioinformatics study reported a reduction in CTL recognition as a result of A3G mutation in epitopes (119). On the other hand, it was demonstrated that increasing the turnover of truncated HLA-restricted peptides, generated due to the action of A3G, can enhance the CTL response in a mouse model of CTL responses to HIV (120). We previously measured CTL recognition of wild-type or A3G-mutated epitopes *ex vivo* by CTL from HIV-infected individuals. We considered a limited subset of CTL epitopes known to elicit CTL recognition, and we focused on A3G-induced mutations in epitope residue positions 3, 5, and 7, which would mainly impact TCR recognition. We found that in the vast majority of instances, A3G-induced mutations in CTL epitopes abrogated CTL recognition of epitopes in an HLA-dependent manner (114, 117, 118). Moreover, we showed that A3G mutational hotspots are enriched in the viral genomic sequences encoding immunogenic CTL epitopes in Gag, Pol, and Nef (117). This is in agreement with the earlier study that also found enriched A3G hotspot motifs within the rapidly diversifying CTL escape sites in Env (119). Interestingly, and in contrast to our findings on A3G hotspot motif enrichment in CTL-encoding epitopes, another study reported that A3G hotspots are less frequently located at genomic locations encoding for the V1-V5 region, the most variable regions of the gp120 envelope protein, in order to hold in reserve its potential mutational capacity for long-term adaption of HIV to the antibody response (122).

Most of our knowledge about epitope-specific CTL responses in chronic HIV infection comes from studies using the standard IFN-γ ELISpot assay (123). Besides experimental approaches, a variety of computational tools for prediction of mutations and their impact have provided valuable information. Different sets of algorithms such as artificial neural networks (ANN), average relative binding (ARB), stabilizing matrix method (SMM) and others have enabled prediction of CTL epitopes within the viral proteome based on HLA-binding affinity (124, 125). The web algorithm HLA binding predictors have a broad allelic coverage with as much as 90–95% accuracy (124–128). HLA binding and subsequent recognition by TCR are the most selective steps in the peptide presentation pathway (129). However, other processes upstream of HLA binding such as proteasomal cleavage, TAP transporter and the stability of the peptide-HLA complex also shape viral epitope availability (130). Prediction tools, such as NetChop and PaProC have been developed based on protein degradation by purified proteasomes to predict potential cleavage sites (131–134). The reliability of these tools has been shown (135–138).

Thus, mutations that impact protein proteasomal processing and/or epitopes' HLA binding can lead to loss of CTL recognition and immune escape (88, 89, 96, 139, 140), but the extent to which A3G mutations could potentially impact each of the successive stages of CTL epitope generation and presentation is not known. Here we utilized the aforementioned computational tools to construct a comprehensive CTL epitope map of HIV based on the steps of antigen presentation: proteasomal cleavage, TAP transporter efficiency and HLA-binding affinity. We simulated all possible A3G-mediated mutations within and outside CTL epitope-encoding sequences of the HIV genome. We then examined predicted consequences for CTL epitope generation. We also probed whether the positions and predicted consequences of A3G-mediated mutations are random, or rather indicative of co-evolution of the HIV genome with the action of the host mutator. In cases where such co-evolution was observed, we studied the depth and extent of the strategies used by the HIV genome to influence the outcomes of A3G activity. Since our experimental system is devoid of the immense *in vivo* selection pressure for CTL escape, and hence able to predict enhanced generation of CTL epitopes as well as the opposite scenario of immune evasion without bias, the analysis provides a unique lens for considering how viral genomes co-evolve with host restriction factors.

## Materials and Methods

### A3G-Induced Mutation Simulation

Simulation of A3G-induced mutations was carried out as previously described (117). Briefly, the whole genome of the HIV-1 isolate HXB2 BRU was obtained from NCBI. This sequence was chosen since it was used in previous works and model systems that studied the role of A3G on HIV CTL escape (117, 119). A3G-induced mutations (G-to-A) on the 5′-most dG in A3G's trinucleotide hotspot motifs considering the sense of the +ve sense strand (GGG, GGA, and GGT) were manually simulated and translated to amino acid sequence. For this analysis, we considered first-round mutations. For multiple back-to-back A3G hotspots, all possible amino acid alteration consequences of A3G-induced mutations were considered.

### Prediction of CTL Epitopes, HLA Binding, and Proteasomal Cleavage

To generate a comprehensive list of all potential CTL epitopes of HIV, we considered all HIV peptides that are predicted to be efficiently processed by proteasomes and also bind to HLA class I molecules. We identified the portions of the HIV genome encoding known CTL epitopes using the HIV Molecular Immunology Database (http://www.hiv.lanl.gov/content/immunology/tables/ctl_summary.html). We evaluated the predicted MHC binding affinity of wild-type and A3G-mutated variant CTL epitope sequences of Gag, Pol, Env, and Nef restricted to HLA-A02:01, -A03:01, -B57:01, and -B35:01. We utilized epitope prediction algorithms enabling us to investigate the impact of mutations at A3G hotspots within or in flanking regions of the predicted and known epitopes on HLA affinity binding, and epitope processing. Here we used NetMHCpan 4 (http://www.cbs.dtu.dk/services/NetMHCpan/) using artificial networks (ANN) to construct a fine CTL epitope map based on HLA-I binding. The NetMHCpan 4 server predicts binding of peptides to any HLA molecule of a known sequence using ANNs (127, 136, 141). Then the Immune Epitope Databases (IEDB) server (http://tools.iedb.org/processing/) was applied for further prediction based on proteasomal cleavage, TAP transporter efficiency and HLA binding affinity to improve the selection of potential epitopes. To evaluate the impact of A3G alterations on HLA-binding, we only considered predicted epitopes with a high-rank score between 0 and 0.5 percentile as strong HLA binders and 0.5–2.00 percentile as weak HLA binders (136). We then calculated a Delta from the wild-type sequence HLA binding score to evaluate the change in predicted HLA affinity caused by A3G-induced mutation. We set a Delta of 0.1 as a threshold of significant difference for enhanced or diminished HLA binding affinity, based on distribution analysis of the difference values. We used NetChop 3.1 (http://www.cbs.dtu.dk/services/NetChop/) to display the impact of the mutation on proteasomal cleavage. The program C-term 3.0 network is trained with a database consisting of 1260 publicly available HLA class I ligands (using only the C-terminal cleavage site of the ligands). The highest probabilities of cleavage (threshold set at 0.5) were applied based on default program recommendation (142). To predict proteasomal cleavage sites, wild-type and A3G-induced mutated polypeptide of Gag, Pol, Env, and Nef were submitted to NetChop 4.

### Analysis of A3G Hotspot Frequency in Sequences Encoding CTL Epitopes at the Nucleotide Level and Prediction of Amino Acid Alteration Consequences

To investigate the enrichment of A3G hotspots of CTL epitopes Gag, Pol, Env, and Nef restricted to HLA-A2:01, HLA-A3:01, HLA-B57:01, and HLA-B35:01, we counted the number of A3G hotspot motifs (GGA, GGG, and GGT) in CTL epitope-encoding regions vs. non-CTL epitope-encoding sequences. We normalized for gene size by dividing the frequency of hotspots by the total number of analyzed nucleotides in each gene. The normalized hotspot frequencies at the nucleotide levels were calculated for sequences encoding CTL epitopes restricted to each individual HLA, and non-CTL epitope encoding sequences. Then, the ratio of hotspot frequency was determined for inside to outside epitope-encoding sequences, for each A3G hotspot motif and each restricting HLA. As controls to evaluate potential A3G sequence enrichment in sequences encoding CTL epitopes, we conducted a parallel analysis with randomly shuffled HIV genomic sequence using the “Shuffle DNA” function of the Sequence Manipulation Suite (http://www.bioinformatics.org/sms2/shuffle_dna.html) resource. The HIV sequence was randomly shuffled six independent times, and A3G hotspot enrichment analysis was performed for each hotspot motif and restricting HLA, using the same border locations of sequences encoding CTL epitope sequences in the actual HIV genomic sequence. MATLAB was used to describe the distribution of amino acid alterations in epitope-surrounding regions, considering a 32-amino acid boundary around each 8–11 mer epitope, with a limit of either 2 or 4 A3G-induced amino acid changes on either the N- or C-terminal sides of each CTL epitope within this boundary considered to be a clustered pattern. Graphpad Prism 5 was used to generate the schematic graph to display the distribution of A3G-induced mutation within and in the flanking regions of CTL epitopes Gag, Pol, Env, and Nef.

For analysis of amino acid alteration consequences of A3G enrichment in CTL-encoding sequences, affected amino acids (as a result of non-synonymous A3G-mediated mutations), or non-affected amino acids (as a result of silent A3G-mediated mutations) were determined, and frequencies of amino acid consequences inside or outside epitope-encoding sequences were normalized to the total amino acid number of regions of each polypeptide (Gag, Pol, Env, and Nef) present in epitope or non-epitope regions, for each restricting HLA. Then, the ratio of normalized non-synonymous to silent substitutions in CTL epitope encoding region to non-CTL epitope encoding region was calculated for A3G-induced mutations of Gag, Pol, Env, and Nef, restricted to each HLA. The ratio of non-synonymous and silent A3G-mediated amino acid substitutions in the CTL epitope encoding region to non-CTL epitope encoding region was calculated in two ways for each individual A3G hotspot motif. First, the ratio of A3G-induced mutations resulting in non-synonymous residue changes to silent mutations inside CTL epitopes was divided by the same ratio determined for regions of each polypeptide located outside CTL epitopes. Second, the frequency of A3G-mediated non-synonymous mutations inside CTL epitopes was divided by the frequency of A3G-mediated non-synonymous mutations outside CTL epitopes, ignoring silent consequences.

To analyze the distribution pattern of A3G hotspot positioning in the HIV genome, we divided the entire length of the genome into 60 bp stretches and counted the number of A3G hotpots whose mutation would result in non-synonymous amino acid changes or stop codon generation. This pattern was plotted and compared against the known mutational gradient of the HIV genome as previously described (35, 143). In the same manner, to analyze the distribution pattern of A3G hotspots whose mutation is predicted to result in CTL escape, we considered, for each HLA, A3G hotpots that fall in CTL epitope-encoding sequences and whose mutation caused a predicted decrease in HLA binding affinity, as described above. For each HLA, we also generated a map of the positions of CTL epitope-encoding sequences across the entire HIV genome. We derived a map of normalized escape potential which we defined as the number of CTL-escape inducing A3G hotspots in each 60 bp segment, normalized (divided) by the total number of A3G hotspots within the segment. Based on the normalized escape potential and the number of CTL epitopes encoded in each 60 bp segment, for each HLA, we derived a map of escape factor which we defined as the product of the number of CTL epitopes and normalized escape potential. Thus, the escape factor value represents the potential for A3G-induced mutations to generate CTL-escape in any given 60 bp increment of the HIV genome.

## Results

### Potential Wild-Type and A3G-Mutated CTL Epitopes in Gag, Pol, Env, and Nef

We utilized NetMHCpan 4 and IEDB using ANN to construct a CTL epitope map based on proteasomal cleavage, TAP transporter efficiency and HLA-binding affinity. Using entire peptide sequences Gag, Pol, Env, and Nef, we generated a list of all potential HLA-binding peptide epitopes. Although the binding affinity data covers 172 HLA molecules (136), we restricted our analysis to HLA-A02:01, -A03:01, -B57:01, and -B35:01 because HLA-A02:01 and A03:01 are frequent in the population, and HLA-B57:01 and -35:01 correlate with robust and weak HIV-specific CTL responses, respectively. Based on the potential definition of all possible epitopes through the presence of HLA-binding anchor residues in the HIV proteome, the number of all putative potential epitopes is significantly higher than those that actually elicit CTL responses, due to limitation in either epitope processing, presentation to TCR, and the many complex physiological and immune response dynamics that underlie the CTL response that cannot be accounted for by epitope prediction algorithms (144). Nevertheless, we noted that the set of epitopes that we generated on the bases of HLA binding and proteasomal processing predictions included the majority (~70%) of experimentally-verified CTL epitopes listed in the HIV Molecular Immunology Database (Table S1). In addition to the predicted set of epitopes, we also included in our analyses experimentally-known CTL epitopes.

**Figure 1:**
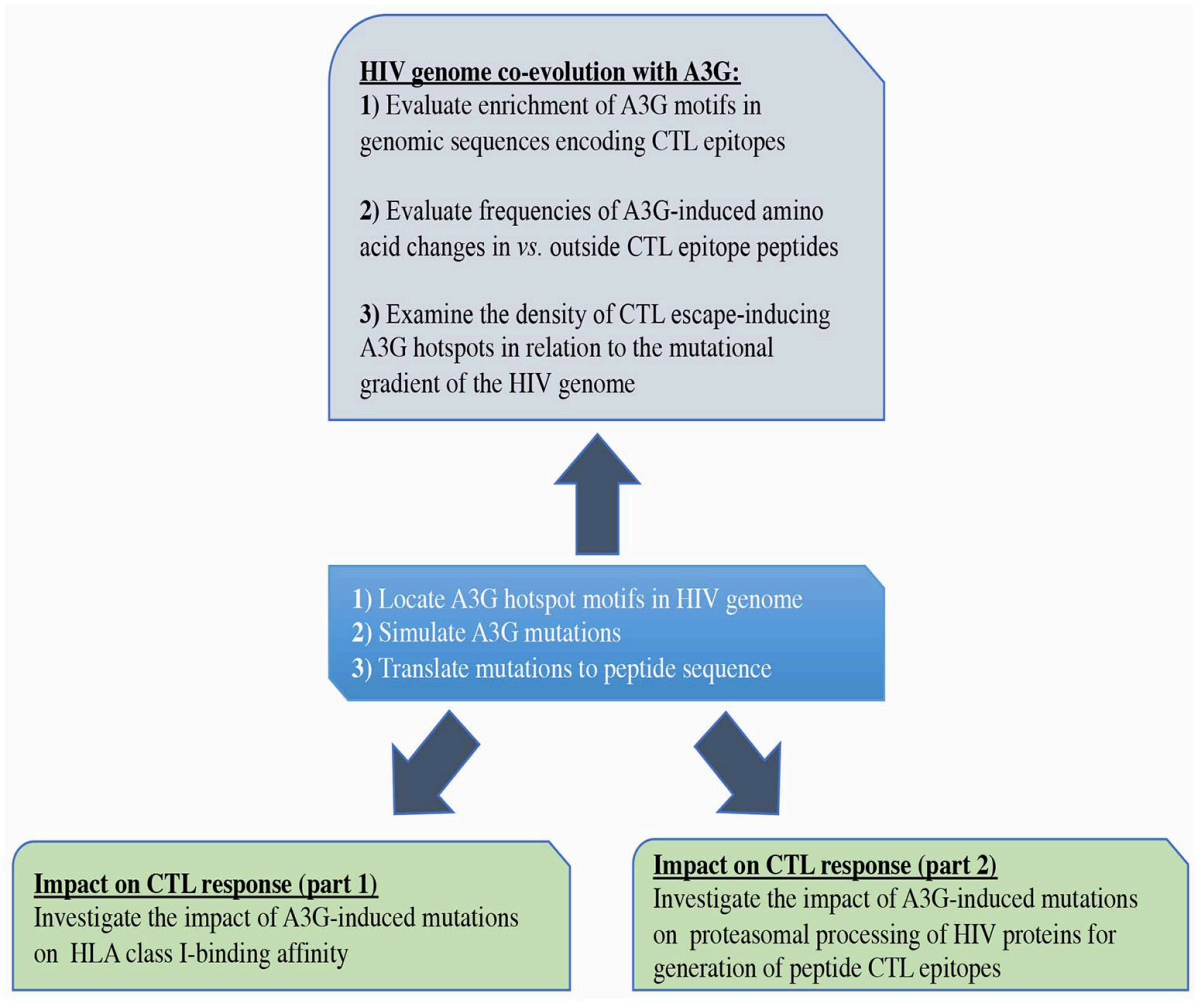
Study design. First, we predicted all possible CTL epitopes of Gag, Pol, Env, and Nef restricted to HLA-A2:01, HLA-A3:01, HLA-B57:01, and HLA-B35:01, simulated A3G-mediated mutations in HIV genomic sequences encoding these epitopes and translated these into peptide mutations (central box). We evaluated the potential impact of A3G-induced mutations on generation of CTL epitopes (lower boxes). We also examined whether potential for CTL escape has left an evolutionary imprint on the HIV genome by studying the pattern and distribution of A3G hotspots across the HIV genome, in the context of their potential for facilitating the generation of CTL escape mutants (Top box).

Thus, we generated an epitope list which includes all potential CTL epitopes restricted by HLA-A2:01, -A3:01, -B57:01, and -B35:01. In total, for Gag, Pol, Env, and Nef, we identified 14-12-14-10, 19-33-26-21, 22-14-20-8, and 8-6-9-8 epitopes restricted to HLA-A2:01, -A3:01, -B57:01, and -B35:01, respectively (Table 1 and Table S1). To dissect the role of A3G at each step of CTL epitope generation, we simulated A3G mutations in sequences encoding Gag, Pol, Env, and Nef. A3G mutations (G-to-A) on the 5′-most dG in A3G hotspot trinucleotide motifs (GGG, GGA, and GGT) were simulated and translated to the peptide sequence (Figure 1). We found 16-16-21-13 (Pol), 8-10-11-7 (Gag), 5-2-6-5 (Nef), and 13-8-15-3 (Env) restricted toHLA-A2:01, -A3:01-, -B57:01-, and -B35:01-, respectively, whose encoding sequences contain A3G hotpots. After simulation of A3G-induced mutations at these motifs, we identified 33-33-44-20 possible mutated epitopes restricted to HLA-A2:01, -A3:01, -B57:01, and -B35:01 for Pol, of which 25 alterations include stop codons (Table 1 and Table S1). These numbers were 8-20-22-8 and 12 stop codons for Gag, 11-4-14-15 and 9 stop codons for Nef, 28-20-31-6, and 29 stop codons for Env. These results indicate that A3G-induced mutations can potentially alter CTL epitopes restricted to all four examined HLAs in Gag, Pol, Env, and Nef (Table 1 and Table S1). Considering all predicted wild-type epitopes, Gag, Pol, Env, and Nef contained 21, 40, 26, and 12% of the predicted CTL epitopes respectively. 25, 30, 26, and 19 of all CTL epitopes were restricted to HLA-A2:01, -A3:01, -B57:01, and -B35:01, respectively. After simulating A3G-induced mutations, 22, 42, 25, and 11% of all mutated epitopes came from Gag, Pol, Env, and Nef and 26, 23, 33, 18% of all epitopes were restricted to HLA-A2:01, -A3:01, -B57:01, and -B35:01, respectively. This initial comparison between the distribution of wild-type vs. A3G-mutated CTL epitopes suggested a bias for A3G-mediated mutations in HLA-B57-restricted epitopes, consistent with previous suggestions for a role of A3G in mediating decreased CTL recognition for peptides restricted to protective HLAs such as B57 (117, 118).

**Table 1:** Summary of the number of potential CTL epitopes restricted to HLA-A2:01, HLA-A3:01, HLA-B57:01, and HLA-B35:01 for wild-type and A3G-mediated mutated Gag, Pol, Env, and Nef proteins.

**Hotspot**	**Wild type**	**Epitope containing hotspot**	**Altered**	**Altered containing stop codon**
**NUMBER OF HLA-A2:01, -A3:01, -B57:01, and -B35:01-RESTRICTED EPITOPES IN Gag**				
**Gag**				
**HLA2:01**		
	14	8
GGG			1	0
GGA			6	2
GGT			1	1
Total			8	3
**Gag**				
**HLA-A3:01**		
	12	10
GGG			10	1
GGA			7	0
GGT			3	0
Total			20	1
**Gag**				
**HLA-B57:01**		
	14	11
GGG			6	0
GGA			15	6
GGT			1	1
Total			22	7
**Gag**				
**HLA-B35:01**		
	10	7
GGG			3	1
GGA			3	0
GGT			2	0
Total			8	1
**NUMBER OF HLA-A2:01, -A3:01, -B57:01, and -B35:01-RESTRICTED EPITOPES IN Pol**				
**Pol**				
**HLA2:01**		
	19	16
GGG			10	1
GGA			15	4
GGT			4	0
Total			33	5
**Pol**				
**HLA-A3:01**		
	33	16
GGG			16	3
GGA			15	0
GGT			2	0
Total			33	3
**Pol**				
**HLA-B57:01**		
	26	21
GGG			16	4
GGA			22	10
GGT			6	1
Total			44	15
Total			20	2
**Pol**				
**HLA-B35:01**		
	21	13
GGG			4	0
GGA			11	1
GGT			5	1
**NUMBER OF HLA-A2:01, -A3:01, -B57:01, and -B35:01-RESTRICTED EPITOPES IN Env**				
**Env**				
**HLA2:01**		
	22	13
GGG			7	0
GGA			17	3
GGT			4	1
Total			28	4
**Env**				
**HLA-A3:01**		
	14	8
GGG			5	0
GGA			13	3
GGT			2	0
Total			20	3
**Env**				
**HLA-B57:01**		
	20	15
GGG			12	4
GGA			14	12
GGT			5	3
Total			31	19
**Env**				
**HLA-B35:01**		
	8	3
GGG			0	0
GGA			6	3
GGT			0	0
Total			6	3
**NUMBER OF HLA-A2:01, -A3:01, -B57:01, and -B35:01 RESTRICTED EPITOPES IN Nef**				
**Nef**				
**HLA2:01**		
	8	5
GGG			5	0
GGA			6	2
GGT			0	1
Total			11	3
**Nef**				
**HLA-A3:01**		
	6	2
GGG			2	0
GGA			2	0
GGT			0	0
Total			4	0
**Nef**				
**HLA-B57:01**		
	9	6
GGG			7	0
GGA			4	2
GGT			3	2
Total			14	4
**Nef**				
**HLA-B35:01**		
	8	5
GGG			9	0
GGA			6	1
GGT			0	1
Total			15	2

### The Potential Impact of A3G-Induced Mutations on HLA Binding Affinity

To examine the specific impacts of A3G-induced mutations on HLA-binding, we considered predicted epitopes with a high-rank NetMHCpan 4 score between 0 and 0.5 percentile as strong HLA binders and 0.5–2.0 percentile as weak HLA binders, according to default parameters of the prediction algorithm (136) (Figure 1, Table S1). However, we noted that 30% of experimentally-verified epitopes exhibit out of range and low HLA binding affinity scores; hence, their aforementioned absence in the total predicted pool of CTL epitopes (Table S1). To evaluate the change in predicted HLA affinity that occurred as a result of each A3G-induced mutation, we calculated a Delta value from the wild-type sequence HLA binding score. We set 0.1 as a threshold of difference for increased or reduced HLA binding affinity because below this value poor correlation was observed between the predicted HLA affinity rank and absolute nM affinities.

**Figure 2:**
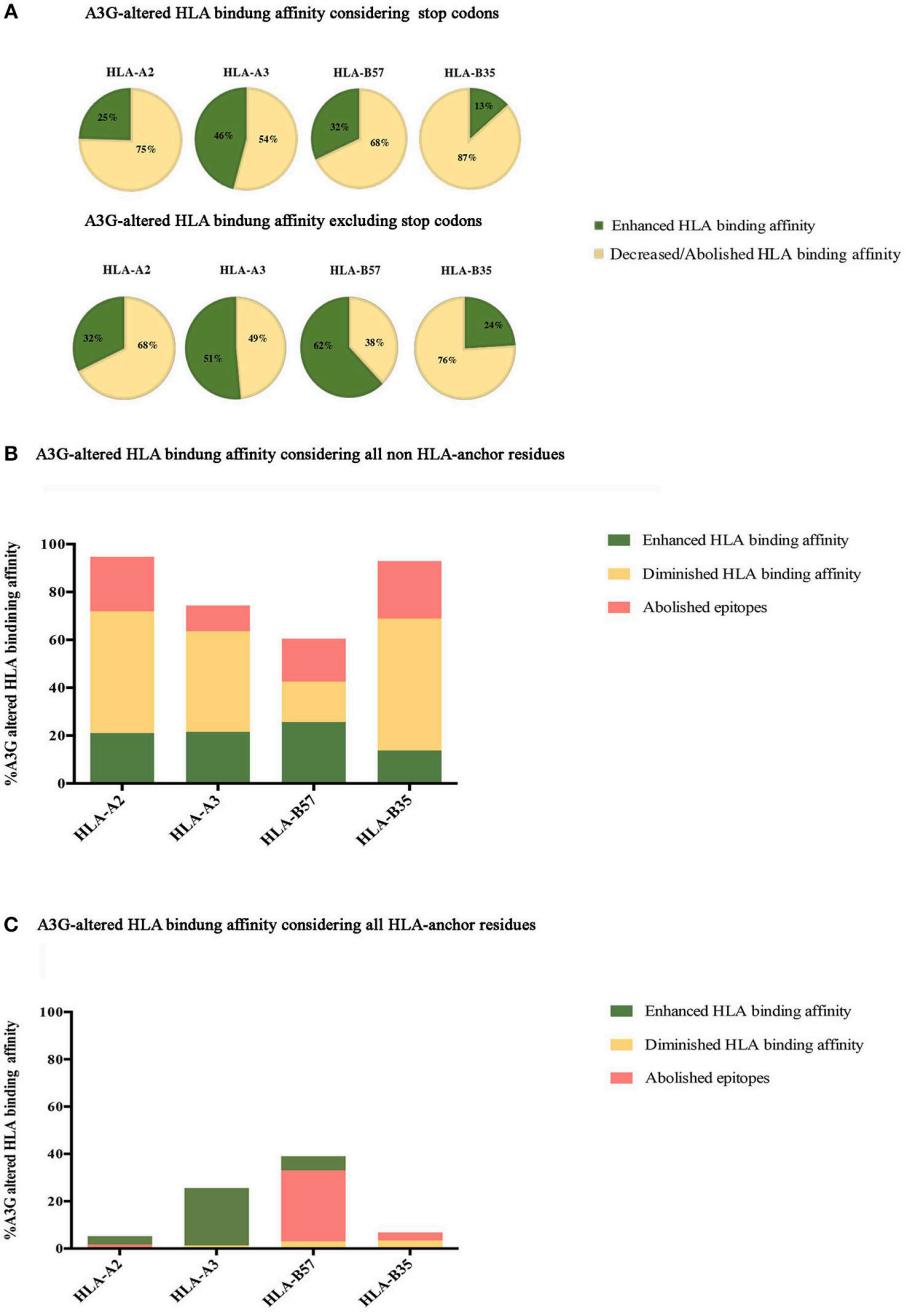
Impact of A3G-induced mutations on HLA-binding. The impact of A3G-mediated mutations on HLA-binding affinity was predicted using NetMHCpan 4 to examine epitopes restricted to HLA-A2:01, HLA-A3:01, HLA-B57:01, and HLA-B35:01 for Gag, Pol, Env, and Nef proteins. Epitopes within the range of strong and weak HLA-binding affinity were considered as potential CTL epitopes. Delta value between A3G-mutated wild-type epitope scores were calculated with 0.1 rank difference as a threshold of meaningful difference for increased or reduced HLA binding affinity. **(A)** The impact of all A3G induced-mutations on HLA binding of epitopes in Gag, Pol, Env, and Nef restricted to HLA-A2:01, HLA-A3:01, HLA-B57:01, and HLA-B35:01 considering stop codon (top panel) and excluding stop codon (bottom panel). **(B)** The specific impact of A3G induced-mutations at non-anchor residues on HLA binding. **(C)** The specific impact of A3G-induced mutations on HLA-binding affinity considering only mutations at the N-terminal anchor position (amino acid residue 2 in a 8-11 mer) and the most C-terminal HLA anchor residue.

Although A3G-induced stop codons would not lead to infectious virus production, viral genomes containing stop codons can produce immunogenic truncated peptides which contain CTL epitopes (120). Thus, we considered all A3G-mutations, including stop codon generators (Figure 2A, top panel), or excluding stop codons (Figure 2A, bottom panel). Considering all A3G-induced mutations, 25, 46, 32, and 14% of HLA-A2:01-, -A3:01-, -B57:01-, and -B35:01-restricted epitopes exhibited increased HLA-binding affinity after A3G simulation mutation. Conversely, 75, 54, 68, and 86% of HLA-A2:01-, -A3:01-, -B57:01-, and -B35:01-restricted epitopes exhibited decreased HLA-binding affinities as a result of A3G-induced mutations (Figure 2A, top panel). Excluding A3G-mediated stop codons, 32, 51, 62, and 24% of HLA-A2:01-, -A3:01-, -B57:01-, and -B35:01-restricted epitopes increased HLA-binding affinity after A3G simulation mutation, whilst 68, 49, 38, and 76% of A3G-induced mutations HLA-A2:01-, -A3:01-, -B57:01-, and -B35:01-restricted epitopes led to decreased HLA-binding affinities (Figure 2A, bottom panel).

Next, we sought to break down the effect of A3G-induced mutations at epitope-HLA anchor vs. non-anchor residues. A3G-induced mutations in non-anchor residues were predicted to lead to enhanced, diminished, or abolished HLA binding affinity for epitopes restricted to all 4 HLAs, with diminished/abolished HLA binding being the most common predicted outcome for HLA-A2:01-, -A3:01-, - and -B35:01-restricted epitopes (74, 53, 80% of all A3G mutations, respectively); for HLA-B57:01 however, that outcome was nearly equal to potential for enhanced HLA affinity (35%) (Figure 2B). Escape mutations that reduce class I HLA binding commonly occur at the N-terminal (amino acid position 2 in the peptide epitope) and/or the C terminal (e.g., amino acid position 9 in a 9mer peptide epitope) anchor residues in most epitopes (84, 96, 145). Thus, we then considered A3G-induced mutations that target the anchor residues. A3G-induced mutations in anchor residues were predicted to lead to enhanced, diminished or abolished HLA binding affinity for epitopes restricted to all 4 HLAs, with diminished/abolished HLA binding being the most common predicted outcome for -B57:01-, - and -B35:01-restricted epitopes (33, 7% of all A3G mutations, respectively). In contrast, for HLA-A2:01- and -A3:01, A3G-induced mutations at anchor residues mediated diminished/abolished HLA binding affinity with the same or much lower levels than enhanced HLA binding affinities (2 and 1%, respectively) (Figure 2C).

Considering all epitopes that were within the aforementioned HLA-binding threshold range, 2 and 23% of A3G-induced amino acid changes targeted the N and C-terminal anchor positions, respectively. Twenty-three percent is approximately two-fold higher than expected by random chance (84, 146): if A3G-induced mutations were equally distributed amongst all residues in a pool of 8–11 mer peptides, then each residue ought to have a ~10% probability of being targeted. In total, A3G can potentially generate a stop codon in 30% of all epitopes that contain A3G hotspots. Of these, 43% led to a stop codon at the most C-terminal position, of which the overwhelming majority (92%) were restricted to HLA-B57:01, since tryptophan (the TGG codon) is the C-terminal anchor for HLA-B57:01 (Figure 2A, compare top and bottom panels). This codon, which has a high likelihood of containing an A3G hotspot motif dependent on the next downstream nucleotide, is the most susceptible codon for generating a stop due to G-to-A mutation (37, 38). Also, we observed 30% of all potential A3G-induced mutations in CTL epitopes were located at residue positions 3, 5, and 7 which are key for TCR recognition (147), while 40% of A3G-mediated substitutions targeted residue positions 1, 4, 6, 8, 9, and 10. These results indicate that the N- and C-terminal anchor residues are under- and over-targeted by A3G for mutation, whilst the middle positions are apparently equally targeted. Furthermore, the increased A3G targeting of the most C-terminal anchor residue reflects its overwhelming propensity for stop codon generation in HLA-B57:01-restricted epitopes (Figure 2A). Based on these analyses, we conclude that A3G-induced mutations can increase or decrease HLA-binding affinities of potential CTL epitopes; however, the major outcome considering all mutations (non-synonymous amino acid changes and stop codons) at all residues (anchor and non-anchor) was decreased HLA-binding affinity. These results are consistent with previous observations that the CTL-epitope-encoding sequences of HIV have evolved to channel A3G-induced mutations to mediate CTL escape. On the other hand, we also observed the generation of 18 neo-epitopes based on enhanced HLA-binding affinity.

### The Role of A3G-Induced Mutations on Proteasomal Processing of Epitopes

We utilized NetChop 3.1 to examine the impact of A3G-induced mutations on the proteasomal processing of HIV proteins, which is the step before peptide epitope generation for HLA binding. We submitted the entire sequences of Gag, Pol, Env, and Nef to NetChop, either in wild-type format, or including all possible A3G-induced mutations. On the entire peptide sequence, we overlaid the map of HLA-A2:01, -A3:01-, -B57:01, and -B35:01-restricted CTL epitopes. Each residue within wild-type or A3G-mutated Gag, Pol, Env, and Nef proteins was then assigned a cleavage prediction score (default threshold of 0.5 is considered a cleavage site) (142), and scores at each residue position were compared between wild-type and A3G-mutated proteins (Supplementary File 1: excel table). For this analysis, we considered two categories of A3G-induced mutations: those that fell within individual CTL epitopes, or those that fell outside but within six amino acids upstream or downstream of the N-or C- terminal residues of the epitope (148). A3G-induced mutations that generated new/enhanced cleavage sites within a CTL epitope, or abolished/decreased cleavage within the six amino acids adjacent to the epitope would likely lead to diminished proteasomal processing of the epitope. Conversely, A3G mutations that enhanced cleavage in the adjacent region of an epitope or abolished/decreased cleavage within the epitope itself would likely lead to enhanced proteasomal processing of the epitope.

In this manner, we quantified the impact of A3G-induced mutations on CTL epitope proteasomal cleavage (Supplementary File 1, Table 2). Considering all A3G-mediated mutations, for epitopes restricted to HLA-A2:01, -A3:01-, -B57:01, and -B35:01, respectively, 42, 53, 43, 45% of all A3G-induced mutation events resulted in decreased predicted proteasomal processing, whilst 58, 47, 57, and 55% of mutations resulted in generation of sites predicted to enhance proteasomal processing. If epitopes were categorized by protein of origin rather than restricting HLA, for Gag, Pol, Env, and Nef, respectively, 54, 43, 41, and 53% of all A3G-induced mutations resulted in decreased predicted proteasomal processing, whilst 46, 57, 59, and 47% resulted in enhanced predicted processing. Excluding A3G-mediated stop codon generation, for epitopes restricted to HLA-A2:01, -A3:01-, -B57:01, and -B35:01, respectively, 43, 56, 53, and 43% of all A3G-induced mutations resulted in decreased predicted proteasomal processing, whilst, 57, 44, 43, and 57% resulted in enhanced proteasomal processing.

**Table 2:** Proteasomal cleavage site prediction using NetChop 3.1 for Gag, Pol, Env, and Nef for HLA-A2:01, HLA-A3:01, HLA-B57:01, HLA-B35-restricted epitopes.

**Protein**	**HLA-A2:01**						**HLA-A3:01**						**HLA-B57:01**						**HLA-B35:01**					
	**New sites**		**Eliminated sites**				**New sites**		**Eliminated sites**				**New sites**		**Eliminated sites**				**New sites**		**Eliminated sites**			
	**Inside CTL epitope**	**Outside CTL epitope**	**Inside CTL epitope**		**Outside CTL epitope**		**Inside CTL epitope**	**Outside CTL epitope**	**Inside CTL epitope**		**Outside CTL epitope**		**Inside CTL epitope**	**Outside CTL epitope**	**Inside CTL epitope**		**Outside CTL epitope**		**Inside CTL epitope**	**Outside CTL epitope**	**Inside CTL epitope**		**Outside CTL epitope**	
			**Non-stop codon**	**Stop codon**	**Non-stop codon**	**Stop codon**			**Non-stop codon**	**Stop codon**	**Non-stop codon**	**Stop codon**			**Non-stop codon**	**Stop codon**	**Non-stop codon**	**Stop codon**			**Non-stop codon**	**Stop codon**	**Non-stop codon**	**Stop codon**
Gag	7	9	3	2	2	1	11	3	3	0	4	1	9	6	3	4	1	1	3	1	3	1	2	2
Pol	6	10	6	4	2	5	9	14	5	3	10	5	10	10	3	11	5	4	4	10	6	2	3	2
Env	9	10	3	4	4	1	5	6	3	3	2	3	6	8	2	15	5	5	4	6	1	2	1	2
Nef	1	3	1	1	3	0	3	1	3	0	3	1	3	3	3	1	2	1	4	2	3	0	3	0

In total, including A3G-mediated stop codon generation 54% of all A3G-induced mutations that could potentially impact proteasomal cleavage were predicted to lead to enhanced CTL epitope production, whilst 46% could potentially decrease CTL epitope production. These numbers are 51 and 49%, respectively, whist A3G-mediated stop codon generation is excluded (Table 2). These results indicate that there has not been a strong evolutionary pressure maintained on the viral genome for utilizing A3G toward CTL escape at the level of modulating proteasomal processing for CTL epitope generation.

We then investigated whether mutations mediated by A3G are clustered around the CTL epitopes, with more A3G hotspots being present either near the N- or C-terminal boundaries of epitopes, than expected at random. Considering a limit of 4 mutational A3G hotspots (A3G hotspots whose mutation would lead to non-synonymous substitutions) in 32 residues around each epitope, there appeared to be a marked paucity of such clustering (Table 3, Figure S1); however, when this limit was lowered to 2 mutational A3G hotspots, instances of clustering expectedly rose to 50–70% of epitopes. When all A3G hotspots were considered, this clustering proportion rose to 70–80%; thus, if non-mutational A3G hotspots can alter aspects of epitope production pre-translation (e.g., splicing, expression, etc.) this could be considered a significant trend.

**Table 3:** Enrichment of A3G hotspot motifs in flanking regions of sequence encoding CTL epitopes restricted to HLA-A2:01, HLA-A3:01, HLA-B57:01, and HLA-B35:01 for Gag, Pol, Env, and Nef.

**Protein**	**HLA**	**All instances epitope (surrounded by 2 mutations) (%)**	**All instances epitope (surrounded by 4 mutations) (%)**	**Non-synonymous mutation (surrounded by 2 mutations) (%)**	**Non-synonymous mutation (surrounded by 4 mutations) (%)**
Gag	HLA-A2:01	69	7.6	61	0
	HLA-A3:01	53	0	30	0
	HLA-B57:01	78	7.1	64	0
	HLA-B35:01	80	10	50	0
Pol	HLA-A2:01	42	0	31	0
	HLA-A3:01	66	12	45	6
	HLA-B57:01	44	12	29	4
	HLA-B35:01	50	10	35	0
Env	HLA-A2:01	54	14	40	9
	HLA-A3:01	53	13	46	13
	HLA-B57:01	57	15	42	11
	HLA-B35:01	62	25	50	25
Nef	HLA-A2:01	75	12.5	75	0
	HLA-A3:01	83	33	60	0
	HLA-B57:01	75	25	50	0
	HLA-B35:01	75	37	50	12.5

### Patterns and Consequences of A3G Hotspot Distribution Within or Outside CTL Epitope-Encoding Regions

**Figure 3:**
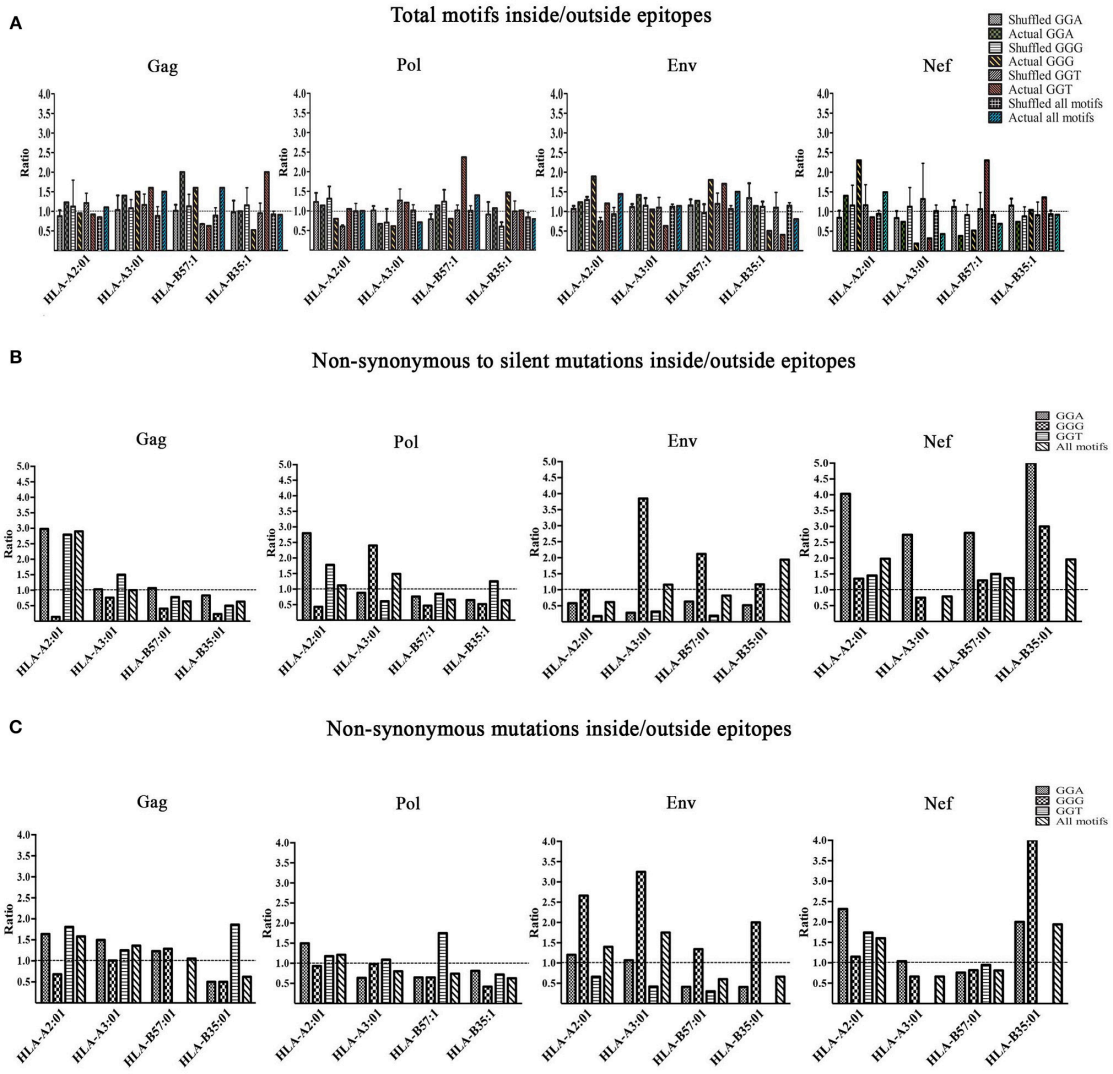
Enrichment of A3G hotspot motifs in CTL-encoding sequences at the nucleotide level and consequences for amino acid substitutions in epitopes. **(A)** Frequency of A3G hotspot motifs inside vs. outside CTL-epitopes restricted to HLA-A2:01, HLA-A3:01, HLA-B57:01, and HLA-B35:01 in Gag, Pol, Env, and Nef proteins. For Gag, Pol, Env, and Nef genes, the number of A3G hotspots inside vs. outside CTL epitope-encoding sequences were counted. The frequency of A3G hotspots (GGG, GGA and GGT) was then calculated by normalizing to total nucleotide length analyzed, in genomic sequences that either encode (inside) or do not encode (outside) CTL epitopes. Thus, an inside: outside ratio of >1 indicates enrichment of A3G hotspots in sequences encoding CTL epitopes. As controls for this evaluation, we included parallel sequences in which the HIV genome was randomly shuffled using “Shuffle DNA” (http://www.bioinformatics.org/sms2/shuffle_dna.html) resource 6 independent times. Retaining the same CTL epitope-encoding/non-encoding genomic positions, we repeated the analyses for the shuffled sequences. **(B)** A3G-induced mutations at the nucleotide level were translated to amino acid sequence changes, for CTL epitopes of Gag, Pol, Env, and Nef restricted to HLA-A2:01, HLA-A3:01, HLA-B57:01, and HLA-B35:01. We counted the number of non-synonymous substitutions as well as silent consequences within and outside CTL epitopes and normalized for total amino acid protein length, followed by calculating a ratio to measure whether A3G-driven non-synonymous substitutions are more frequent than silent ones within vs. outside CTL epitopes. **(C)** A ratio of non-synonymous substitutions inside vs. outside CTL-epitopes restricted to HLA-A2:01, HLA-A3:01, HLA-B57:01, and HLA-B35:01 in Gag, Pol, Env, and Nef proteins.

We then investigated the enrichment of A3G hotspots (GGA, GGG, and GGT) inside vs. outside genomic sequences encoding CTL epitopes restricted to HLA-A2:01, HLA-A3:01, HLA-B57:01, and HLA-B35:01 in Gag, Pol, Env, and Nef genes (Table S2, Figure 3). First, we normalized for total nucleotide length of each gene and calculated the ratio of normalized A3G hotspot frequencies inside to outside epitope-encoding sequences for each protein's CTL epitopes. Thus, an inside: outside ratio >1 would be indicative of A3G hotspot enrichment in CTL-encoding sequences. As a control, we subjected the entire HIV genomic sequence to a random shuffling process, six independent times, but retained the positions/borders of the CTL epitope-encoding sequences. We then conducted the same analysis and expectedly arrived at ratios of ~1 (Figure 3A). We did not observe a generalizable trend of hotspot enrichment (ratio >1) in CTL-encoding sequences; however, when compared to the hypothetical ratio of 1 and the randomly shuffled control analyses, we noted that sequences encoding epitopes restricted to HLA-A2:01 and HLA-B57:01 often exhibited the highest enrichment ratios of 2-2.5 for at least 1-2 out of the 3 A3G hotspot motifs (Table S2, Figure 3A). These data are consistent with our previous observations that viral genomic sequences encoding more immunogenic CTL epitopes (restricted to more common HLA alleles, or those that elicit a more effective CTL response) have evolved to maintain A3G hotspots. Since A3G-mediated mutations which lead to stop codon generation would most often lead to non-infectious genomes, it is difficult to envision how maintenance of such A3G motifs to alter CTL epitopes at the cost of producing a non-infectious virus could be advantageous for the virus. Thus, in our enrichment analyses which was conducted to measure the extent to which the viral genome has evolved to utilize A3G toward CTL escape, we excluded A3G hotspots that would lead to stop codons. Rather, we considered these separately by examining the frequency and positional distribution of stop codon-generating A3G motifs in Gag, Pol, Env, and Nef. We found that between 18 and 43% of stop codons are positioned in the first quarter of each peptide (Figure S2), and there was a general trend of more frequent A3G-mediated stop codon generation in Pol and Env, as compared to Gag and Nef.

Having examined gene sequence A3G hotspot enrichment, we sought to measure the potential consequences at CTL epitope protein level. To this end, all simulated A3G-induced mutations were translated to protein sequences as described above. Since it is known that A3G can mutate the entire viral genome at low levels, epitopes with multiple hotspots and multiple mutated versions were considered independently. We then quantified A3G-induced non-synonymous and silent substitutions that fell within or outside of CTL epitopes of Gag, Pol, Env, and Nef restricted to HLA-A2:01, HLA-A3:01, HLA-B57:01 and HLA-B35:01. Next, we determined the ratio of A3G-induced mutations that caused non-synonymous residue changes to A3G-induced mutations which resulted in silent mutations within CTL epitopes and divided this by the same ratio determined for regions of each polypeptide that fell outside CTL epitopes. This analysis was carried out for each individual A3G hotspot motif, and as a total for all amino acids affected by A3G mutations within each polypeptide (Table S2 and Figure 3B). Thus, a ratio of >1 would indicate that the genomic sequence of HIV has evolved to channel A3G-induced mutations into amino acid changes, more often within CTL epitope-encoding regions as compared to sequences outside these portions. Indeed, we observed numerous instances of significant preferential channeling (ratios up to 4.5) toward non-synonymous residue changes in CTL epitopes of Gag, Pol, Env, and Nef as a result of A3G-induced mutations (Figure 3B). If we ignored A3G-driven silent consequences and evaluated the ratio of only A3G-mediated non-synonymous mutations inside CTL epitope-encoding regions to A3G-mediated non-synonymous mutations outside CTL epitope-encoding regions, we observed that in 28/48 graphed bars (58% of all measurements) the ratio was ≥ 1, with some ratios in the 2–3 range (Figure 3C). In general, the bias for A3G mutations to translate to non-synonymous rather than silent amino acid mutations was more pronounced for Gag, Pol and Nef as compared to Env, consistent with the former three polypeptides housing the vast majority of HIV's CTL epitopes (Figures 3B,C compared to Figure 3A).

**Figure 4:**
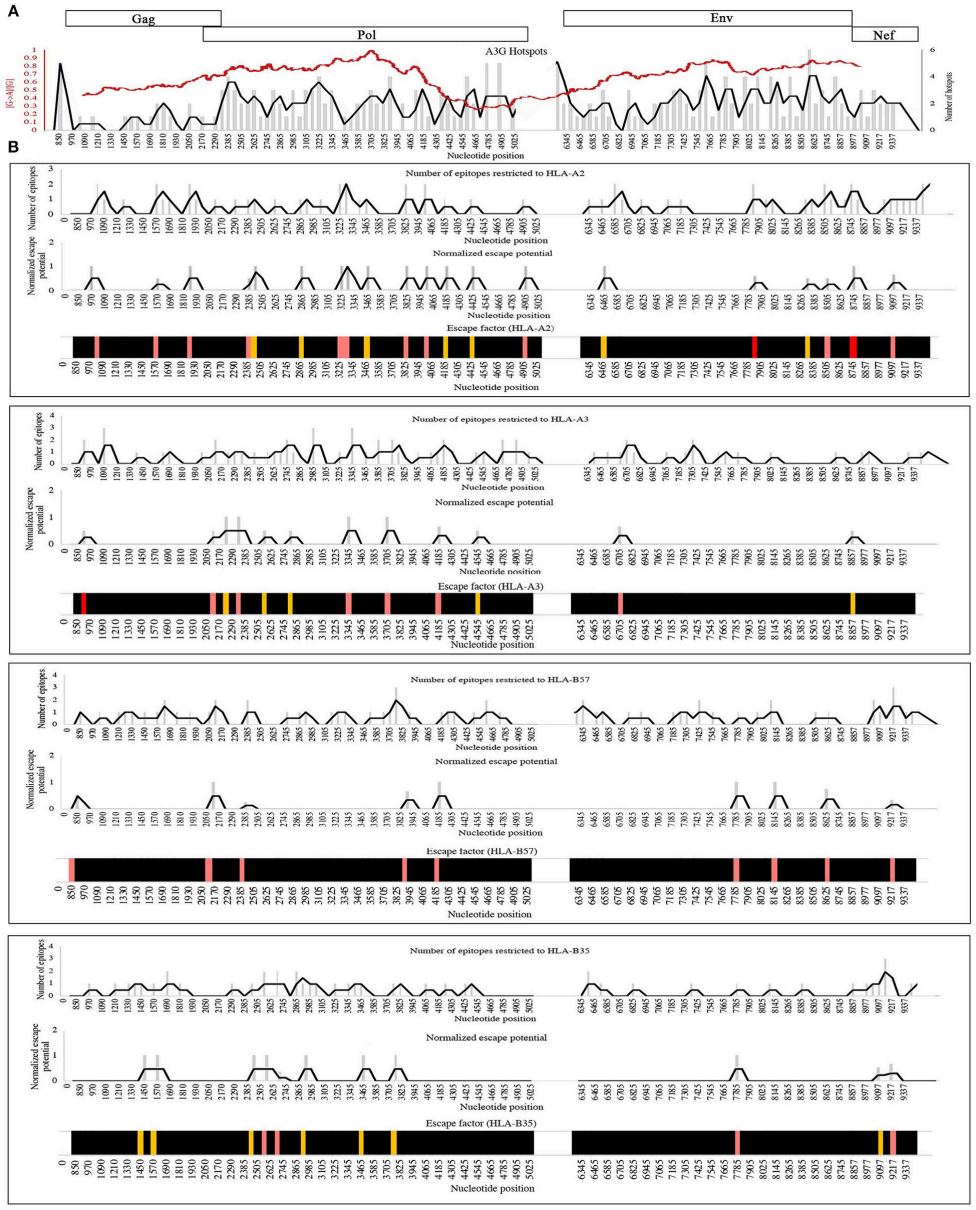
Distribution pattern of CTL escape-inducing A3G hotspots in the context of the entire HIV genome and its mutational gradient. **(A)** To analyze the distribution pattern of A3G hotspots across the HIV genome, we counted the number of A3G hotpots that could result in non-synonymous amino acid changes within windows of 60 bp length across the entire length of Gag, Pol, Env, and Nef. The number of A3G hotspots in each 60 bp window is plotted as gray bars, and the overall pattern is shown as a black line. We then overlaid this pattern against the known mutational gradient of the HIV genome as previously described (red line) (143) (Copy Right License Number: 4460241074666). **(B)** To analyze the distribution pattern of predicted CTL escape-inducing A3G for each HLA, a map of the positions of CTL epitope-encoding sequences for Gag, Pol, Env, and Nef, restricted to HLA-A2:01, HLA-A3:01, HLA-B57:01, and HLA-B35:01 was created (Top panel of each box). A map of normalized escape potential was also generated (middle panel of each box). We defined normalized escape potential as the number of CTL escape-inducing A3G hotspots in each 60 bp segment, normalized (divided) by the total number of A3G hotspots within the segment. Based on the normalized escape potential and the number of CTL epitopes encoded in each 60 bp segment, for each HLA we then constructed a map of escape factor which we defined as the product of the number of CTL epitopes and normalized escape potential (bottom panel). In this map, black indicates no potential for CTL escape (escape factor = 0), orange indicates low potential (escape factor = 1), pink indicates modest potential (escape factor range of 2–4), red indicates high potential (escape factor range of 5–7).

In principle, non-synonymous amino acid changes arising from A3G mutations can enhance or diminish antigen presentation as the proteasomal processing and HLA binding levels (Figure 2, Table 2). To examine the distribution patterns of A3G hotspots that could potentially lead to CTL escape, we generated a map of all A3G hotspots across the entire HIV genome and overlaid this map on the experimentally-determined and well-known gradient of G to A mutations across the HIV genome (Figure 4A). In the context of A3G action, this twin gradient has been suggested to be a consequence of HIV genome's replication dynamics. Certain portions of the HIV minus strand genome remain single-stranded for a longer period compared to other segments, due to dynamics of RNA digestion by RT, the role of the Polypurine tracts (PPT), and subsequent positive sense strand polymerization. These segments are thus more available for A3G targeting resulting in a mutation gradient (34, 35, 143). We observed that regions near the central PPT and the C-terminal end (Nef) that are more highly mutated are rich in A3G hotspots, consistent with the notion that the viral genome has positioned hotspots in genomic locations that are more prone to being targeted by the A3G enzyme (Figure 4A). For each HLA, we plotted the number of CTL-epitope encoding sequences (in Gag, Pol, Env, and Nef) at incremental positions along the entire viral genome length (Figure 4B, top graph of each panel). We also plotted a normalized escape potential graph which represents the likelihood that an A3G hotspot located inside a CTL epitope-encoding sequence can generate a CTL-escape mutation. This was calculated by counting the A3G hotspots predicted to lower HLA binding affinities and normalizing these by the total abundance of A3G hotspots in the given CTL-epitope encoding region (Figure 4B, middle graph of each panel). Considering the number of CTL-epitopes encoded by a given genomic location (top panel), as well as the normalized escape potential of the sequences encoding this epitope (middle panel), we then generated an escape factor map which represents the compound potential for A3G to cause CTL escape across the HIV genome, for each HLA (Figure 4B, bottom graph of each panel). First, we noted that the potential for A3G-generated CTL escape was present throughout the length of the genome, for epitopes restricted to all 4 HLAs; however, it was generally more frequent in regions of the genome with a higher mutational potential and less frequent in regions known to be mutated at lower rates (comparing escape factor maps in Figure 4B to the mutational gradient in Figure 4A). Secondly, epitopes restricted to HLA-A2:01 and HLA-B57:01 exhibited overall higher abundance and frequent positioning of escape-inducing A3G hotspots, with HLA-B57:01-restricted epitopes also containing the highest escape factor values (Figure 4B). Thirdly, regions encoding for Gag, Pol and Nef contained generally a higher density of potentially CTL escape-inducing A3G hotspots, as compared to Env. The polypeptide and HLA-specific patterns observed are consistent with the A3G hotspot enrichment analysis (Figure 3) and taken together suggest the evolution of HIV genome to position A3G hotspot motifs in CTL-encoding regions, and highly mutable regions of the HIV genome, such that they preferentially yield CTL escape-inducing non-synonymous amino acid changes.

## Discussion

Here, we aimed to follow up on previous works suggesting that A3G is a source of CTL escape-inducing mutations. We first mapped all potential CTL epitopes within Gag, Pol, Env, and Nef, and considered the impact of A3G-induced mutations on these epitopes. To this end, we embarked on a two-pronged analysis: first, from the immune recognition perspective, we examined the effect of A3G-induced mutations on the various stages of CTL epitope production, including proteasomal processing and HLA-binding affinities. Second, from the viral genome evolution perspective, we examined whether, where and to what consequence, A3G hotspots have been maintained or enriched in genomic sequences that encode for CTL epitopes. At each stage of all analyses, we considered three individual A3G hotspots (GGA, GGG, and GGT), and potential impact on CTL epitopes restricted to four HLA alleles that have been previously shown to have differential abilities to present immunogenic CTL peptides of HIV. Furthermore, opposite to the notion of CTL escape mediated by A3G-induced mutations, we also considered A3G mutations that can potentially generate novel or more immunogenic CTL epitopes. Despite a wealth of information about the role of A3Gs in CTL escape, knowledge of novel CTL epitopes mediated by endogenous mutators remains poor. We found that although A3G-mediated mutations could potentially enhance or diminish the proteasomal cleavage of Gag, Pol, Env, and Nef into CTL epitopes, the overwhelming impact on HLA binding affinities of CTL epitopes as a result of A3G mutations was decreased affinity.

Here we also provide strong and novel lines of evidence for the co-evolution of the HIV genome with A3G, so as to utilize this host factor toward CTL escape. First, A3G hotspot motifs were positioned in CTL-encoding epitopes so as to preferentially cause non-synonymous mutations. Secondly, most A3G-induced mutations in CTL epitopes resulted in diminished/abrogated HLA binding capacity. Thirdly, the distribution pattern of CTL escape-inducing A3G hotspots across the HIV genome varies with restricting HLAs and generally correlates with the known mutational gradient across the entire HIV genome. These observations shed light on the multiple layers of depth to which the HIV genome has resorted to position A3G hotspots for CTL escape. An earlier study which examined the overall pattern of A3G-mediated non-synonymous vs. silent mutations concluded that A3G has not left an evolutionary footprint on the HIV genome (149). This study broadly examined all A3G/F target motifs but not in the context of a specific biological force which may encourage genome evolution in response to A3G/F. In contrast, we argue that evidence for co-evolution of a pathogen's genome with a host factor may not be broadly apparent but must be sought for in the specific context of the pro/anti-viral biological impacts driven by the host factor and specific locations of the pathogen's genome impacted and under pressure by the host factor action. Thus, we considered HIV genome co-evolution with A3G/F in the context of the potential for CTL escape by focusing on sequences that encode for such epitopes. We find that when considered in this context, there is substantial evidence for the evolution of the HIV genome to subvert the activity of A3G/F toward its own gain. In our analyses of predicted immune response to HIV (HLA binding and proteasomal processing of CTL epitopes) we considered A3G-mediated stop codons; though these would yield non-infectious viruses, truncated proteins that are immunogenic could still be produced (120). On the other hand, we excluded A3G-mediated stop codons from our analyses of viral genome evolution (A3G hotspot enrichment and positioning) since they cannot be considered as an advantageous mode of utilizing A3G to the virus's benefit.

Rather than considering existing A3G hotspots as evidence for their role in selection as we have, the case can also be made for the opposite view; that if the usage of A3G hotspots toward advantageous outcomes for the virus was key, then current sequences circulating at the population level ought to be rather devoid of A3G hotspots and instead rich in the mutated versions. Whilst this argument may hold true in several different contexts, considering it in the context of CTL escape is more difficult. First, CTL escape mutations are usually not broadly selectable at the population level but are highly individual host-dependent since they are intimately connected to HLA genotype. Second, from the virus's perspective, there may be two advantages to maintaining the A3G/F hotspots: first, conflicting demands of replication fitness on one hand and immune evasion on the other, which is best illustrated by the high rates at which certain CTL escape mutations revert to wildtype presumably fitter sequence, especially upon transmission to a new host with a different HLA genotype wherein CTL escape mutations from the previous host are no longer advantageous. Second, it may not be to the advantage of the virus to benefit from maximum CTL escape, as it would limit its replication capacity by quickly eliminating infected host cells (150, 151). Thus, it may be advantageous to conserve some CTL escape potential in the form of A3G hotspots to be available to use when it suits the virus. An example of this very conservation of A3G/F-mutational hotspots has been shown in terms of antibody epitopes in Env (122).

These findings bring to light novel aspects of the interplay between the host mutator A3G and the co-evolution of the viral genome. Overall, A3G-induced mutations were predicted to influence CTL epitope production and HLA binding, both toward the production of more immunogenic epitopes and conversely, toward CTL escape. It is important to consider these results in the context of two additional concepts: first, although the overall action of A3G on the HIV genome is predicted to result more often in CTL escape than in generation of new, more immunogenic epitopes, it is important to note that even if the latter and former occurred with equal probability, the escape mutations would be the dominant outcome under immune pressure *in vivo* (9, 87, 119). Second, in this analysis, we did not take into account the fitness consequences of A3G-induced mutations. Predicting the *in vitro* replicative fitness cost and peptide HLA binding affinity of clinically derived sequences has shown that escape mutations in CTL epitopes of Gag restricted to protective HLA class I alleles carried higher fitness costs and lower levels of reduction in HLA class I binding affinity compared to mutations in epitopes restricted to other HLA class I alleles. This suggests that one way by which protective HLA molecules act is by binding epitopes whose CTL escape mutations incur a high fitness cost with relatively low benefit in terms of HLA-binding affinity reduction (108).

The practical application of this work will lie in determining epitope choice for vaccine design. Epitope clusters and altered epitopes with the potential to be better processed or bound by HLA because of A3G mutations ought to be superior platforms for the development of prophylactic or post-infection CTL-based vaccines. Thus, accounting for and indeed exploiting the action of endogenous genome mutators to design more effective vaccines would represent a strategic advance in HIV vaccine design. In addition, the analyses carried out here should be considered in the context of extensive A3 family enzyme mutations of tumor genomes, as understanding the mechanisms by which a tumor cell can escape, or boost CTL response is critical to developing vaccination and therapies based on CTL epitopes. We and others have postulated that the function of A3 family members in cancer genome mutagenesis may bear parallels to its role in viral genome mutagenesis, as tumor cells are also under pressure to avoid detection by CTL and could use A3-induced mutagenesis to this end (152–159). At present, whether and how frequently this may occur is unknown, and using similar analyses to gain insights will have important implications for the design of personalized anti-tumor CTL-based strategies.

## Author Contributions

FB generated all data, with help from KJ. FB and ML analyzed the data, generated the figures and wrote the manuscript. MG edited the manuscript. We thank our colleague Emma Quinlan for assistance with editing.

### Conflict of Interest Statement

The authors declare that the research was conducted in the absence of any commercial or financial relationships that could be construed as a potential conflict of interest.
