# Supplementary material for: APOBEC3G Regulation of the Evolutionary Race Between Adaptive Immunity and Viral Immune Escape Is Deeply Imprinted in the HIV Genome
Source: Front Immunol. 2019 Jan 11;9:3032. doi: 10.3389/fimmu.2018.03032 (PMC6338068; doi:10.3389/fimmu.2018.03032)
Supplement: Supplementary file 2 [file Data_Sheet_1.PDF]

Supplementary Material

APOBEC3G Regulation of the Evolutionary Race between Adaptive Immunity and Viral Immune Escape is Deeply Imprinted in the HIV Genome

Faezeh Borzooee, Krista D Joris, Michael D. Grant, Mani Larijani*

Immunology and Infectious Diseases Program, Division of Biomedical Sciences, Faculty of Medicine, Memorial University of Newfoundland

*To whom correspondence should be addressed:

Mani Larijani, Division of BioMedical Sciences, Faculty of Medicine, Health Sciences Center, MUN, 300 Prince Phillip Dr., St. John's, NL, Canada, A1B 3V6, mlarijani@mun.ca

Figure S1. Distribution of A3G mutations around potential CTL epitopes. Potential epitope map and A3G simulated mutations for Gag, Pol, Env and Nef polypeptides were overlaid and subjected to GraphPad Prism 5. Distribution of the position of amino acid substitutions (X axis) against the known and predicted epitopes restricted to HLA-A2:01 (purple), -A3:01 (green), -B57:01 (blue), and -B35:01 (green) across **A.** Gag, **B.** Pol **C.** Env and **D.** Nef proteins are shown.

Figure S2. Distribution of potential A3G-induced stop codons. Distribution of stop codon-generating A3G motifs mediated by A3G in Gag, Pol, Env and Nef. **A.** Portion of A3G-mediated stop codons in each quarter of Gag, Pol, Env and Nef. **B.** Distribution of A3G-induced stop codon in Gag, Pol, Env and Nef peptides. **C.** Distribution of A3G-induced stop codon across the Gag, Pol, Env and Nef genomic sequences.

Table S1: Execution of HLA-A2:01, HLA-A3:01, HLA-B57:01 and HLA-B35:01-binding CTL epitopes for wild-type and A3G mutated Pol, Gag, Nef, and Env using NetMHCpan 4 and IEDB, and experimentally-reported CTL epitopes.

Pol											
HLA-A2:01			HLA-A3:01			HLA-B57:01			HLA-B35:01		
Peptide	%Rank<=BindLevel ♦	Total Score§	Peptide	%Rank<=BindLevel ♦	Total Score§	Peptide	%Rank<=BindLevel ♦	Total Score§	Peptide	%Rank<=BindLevel ♦	Total Score§
VLVGPTPVNI<br>VLVRPTPVNI	0.6753 <= WB<br>0.9631 <= WB	-1.31<br>-1.68	VTIKIGGQLK<br>VTIKIRRQLK<br>VTIKIGRQLK<br>VTIKIRGQLK<br>VTIKIKGQLK<br>VTIKIEGQLK	0.4429 <= SB<br>0.9808 <= WB<br>0.5434 <= WB<br>0.5781 <= WB<br>0.5879 <= WB<br>0.7047 <= WB	-1.15<br>-1.35<br>-1.33<br>-1.08<br>-1.28<br>-1.45	SPTRRELQVW	0.0782 <= SB	0.04	FPISPIETV	0.1144 <= SB	-0.9
KMIGGIGGFI<br>KMIRGIGGFI<br>KMIEGIGGFI<br>KMIKGIGGFI<br>KMIGRIGGFI<br>KMIGGIRGFI<br>KMIGGIGSFI	1.4797 <= WB<br>4.0285<br>0.8632 <= WB<br>3.2<br>4.2448<br>1.3189 <= WB<br>0.703 <= WB	-0.56<br>-1.1<br>-0.21<br>-1.06<br>-1.13<br>-5.5<br>-0.11	ILIEICGHK<br>ILIEICRHK	0.5216 <= WB<br>0.4372 <= SB	-0.83<br>-0.84	VTIKIGGQL<br>VTIKIRRQL<br>VTIKIGRQL<br>VTIKIRGQL<br>VTIKIKGQL<br>VTIKIEGQL	0.4416 <= SB<br>0.1630 <= SB<br>0.1171 <= SB<br>0.5089 <= WB<br>0.4299 <= SB<br>0.5643 <= WB	-1.50<br>-1.29<br>-1.18<br>-1.59<br>-1.57<br>-1.50	TVLDVGDAY<br>TVLDVSDAY	0.0455 <= SB<br>0.0348 <= SB	1.31<br>1.6
VLDVGDAYFSV<br>VLDVSDAYFSV	0.9230 <= WB<br>1.0467 <= WB	-1.04<br>-0.91	PAIFQSSMTK	0.2291 <= SB	-0.75	NTPPLVKLW<br>NTPPLVKL*	0.0560 <= SB	-0.54	SPAIFQSSM	0.1035 <= SB	-0.83
SQYALGIIQA<br>SQYALRIIQA	1.8926 <= WB<br>3.4534	-1.86<br>-1.93	FVNTPPLVK	0.0428 <= SB	-1.64	KITTESIVIW<br>KITTESIVI*	0.0681 <= SB	-0.52	VPLDEDFRKY<br>VPLDEDFKKY	0.1239 <= SB<br>0.1442 <= SB	-0.13<br>-0.16
ALTEVIPLT	0.1652 <= SB	-1.11	KLVDFRELNK	0.0570 <= SB	-1.11	ATWIPEWEF<br>AT*IPEWEF<br>ATWIPE*EF	0.2336 <= SB	0.02	EPIVGAETFY<br>EPIVRAETFY	0.1839 <= SB<br>0.5203 <= WB	0.46<br>0.09
IVGAETFYV<br>IVRAETFYV	0.1909 <= SB<br>1.0645 <= WB	-0.09<br>-0.95	GIPHPAGLKK<br>GIPHPARLKK<br>RIPHPAGLKK<br>GIPHPAELKK	0.1265 <= SB<br>0.1896 <= SB<br>0.0333 <= SB<br>0.3557 <= SB	-1.17<br>-1.23<br>-0.36<br>-1.55	TGATVRAACW<br>TGATVKAACW<br>TSATVRAACW<br>TGATVRAAC*	1.4145 <= WB<br>1.3235 <= WB<br>0.2724 <= SB	-0.94<br>-1.04<br>0.11	NPYNTPVF	0.2886 <= SB	-0.39
YELHPDKWTV<br>YELHPDK*TV	0.1931 <= SB	-0.46	IVIWGKTPKFK<br>IVIWRKTPKFK<br>IVI*GKTPKFK	1.6746 <= WB<br>2.1696	-1.31<br>-1.32	KLPIQKETW<br>KLPIQKET*	0.2055 <= SB	-1.14	VPVKLKPGM<br>VPVKLKPRI<br>VPVKLKPGI<br>VPVKLKPRM	0.3105 <= SB<br>2.1076<br>2.56<br>0.2788 <= SB	-1.69<br>-2.83<br>-2.78<br>-1.84
YQLEKEPIV	0.2347 <= SB	-0.23	AIKKKDSTK	0.3534 <= SB	-1.9	VNTPPLVKLW<br>VNTPPLVKL*	0.2427 <= SB	-0.48	IPLTEEAEL	0.3359 <= SB	-0.91
VLPEKDSWTV<br>VLPEKDS*TV	0.2844 <= SB	0.11	KLNWASQIY<br>KLN*ASQIY	0.2175 <= SB	0.66	IQKQGQGQW<br>IQKQGQGQ*	0.3208 <= SB	-1.83	TPPLVKLWY<br>TPPLVKL*Y	0.4578 <= SB	-0.43
KLVGKLNWA<br>KLVRKLNWA<br>KLVEKLNWA<br>KLVGKLN*A	0.4686 <= SB<br>1.8903 <= WB<br>0.1789 <= SB	-0.65<br>-1.28<br>-0.26	TVQPIVLPEK	0.3127 <= SB	-1.18	MGYELHPDKW<br>IGYELHPDKW<br>MSYELHPDKW<br>MGYELHPDK*	0.4018 <= SB<br>0.4109 <= SB<br>0.0497 <= SB	-0.11<br>-0.56<br>0.81	EPVHGVYY<br>EPVHRVYY	0.5059 <= WB<br>1.2853 <= WB	-0.9<br>-1.45
FLWMGYEL<br>FLWIGYEL<br>FLWMSYEL<br>FL*MGYEL	0.5051 <= WB<br>0.4489 <= SB<br>0.9124 <= WB	0.64<br>0.44<br>0.89	KTPKFKLPIQK	0.4168 <= SB	-1.21	WTEYWQATW<br>WTEYWQAT*<br>TEY*QATW	0.4152 <= SB	-0.22	QNPDIVIYQY	0.5211 <= WB	-0.28
YLALQDSGLEV<br>YLALQDSRLE	0.1969 <= SB<br>0.7008 <= WB	-0.77<br>-1.02	ASQIYPGIKVR<br>ASQIYPEIKVR<br>ASQIYPRIKVR	1.3272 <= WB<br>3.00<br>2.5620	-1.6<br>-1.98<br>-1.59	KHQKEPPFLW<br>KHQKEPPFL*	0.4284 <= SB	-1.22	ETPGIRYQY<br>ETPRIRYQY<br>ETPEIRYQY	0.5582 <= WB<br>1.2334 <= WB<br>0.4278 <= SB	-0.62<br>-0.93<br>-0.58
VLFLDGIDKA<br>VLFLDRIDKA	0.2492 <= SB<br>0.7106 <= WB	-1.23<br>-1.6	RMRGAHTNDVK	0.6590 <= WB	-0.75	AIKKKDSTKW<br>AIKKKDSTK*	0.4510 <= SB	-1.77	SPIETVPVKL	0.5839 <= WB	-0.93
HLEGKVILV<br>HLERKVILV	0.2904 <= SB<br>1.0482 <= WB	-1.38<br>-1.81	MTKILEPFRK	0.4907 <= WB	-1.3	PIVLPEKDSW<br>PIVLPEKDS*	0.6233 <= WB	-1.32	VAVHVASGY<br>VAVHVASRY	0.2547 <= SB<br>0.2251 <= SB	0.54<br>0.32
LLWKGEGAV<br>LLWKGERAV<br>LLWKGEKAV<br>LLWKGEEAV<br>LLWKSEGAV<br>LL*KGEGAV	0.4730 <= SB<br>0.3191 <= SB<br>0.2930<= SB<br>0.0772 <= SB<br>1.1560 <= WB	-0.58<br>-0.5<br>-0.47<br>0.14<br>-0.87	QIIEQLIKK	0.2044 <= SB	-1.74	VSAGIRKVLF<br>VSARIKKVLF<br>VSAGIKKVLF<br>VSARIRKVLF	0.3537 <= SB<br>0.5331 <= WB<br>0.2949 <= SB<br>0.6380 <= WB	0.32<br>0.23<br>0.18<br>0.29	KAQDEHEKY	0.4996 <= SB	-0.95
ALVEICTEM<br>ALVEICTEI	0.2316 <= SB<br>0.1256 <= SB	-0.48<br>-0.14	VLFLDGIDK<br>VLFLDRIDK<br>VLFLDEIDK	0.2384 <= SB<br>0.1694 <= SB<br>0.72<= WB	-1.11<br>-0.87<br>-1.57	KTAVQMAVF	0.0496 <= SB	0.88	EPPFLWMGY<br>EPPFLWMSY<br>EPPFLWIGY<br>EPPFL*MGY	0.5941 <= WB<br>0.3445 <= SB<br>0.5941 <= WB	-0.04<br>0.61<br>-0.06
VIYQYMDDLYV<br>VIYQYIDDLYV	2.7296<br>2.7006	-1.75<br>-1.77	DKLVSAGIRK<br>DKLVSARIKK<br>DKLVSAGIKK<br>DKLVSARIRK	0.2665 <= SB<br>0.4782 <= SB<br>0.2904 <= SB<br>0.4538 <= SB	-1.12<br>-1.20<br>-1.33<br>-1.01	KAQDEHEKY	0.2865 <= SB	-1.02	YPGIKVRQL<br>YPRIKVRQL<br>YPEIKVRQL	0.7311 <= WB<br>0.6675 <= WB<br>0.3371 <= SB	-1.94<br>-1.84<br>-1.71

EILKEPVHGV<br>EILKEPVHRV	2.95<br>3.5	-2.35<br>-2.58	VTLTDTTNQK	0.4622 <= SB	-1.22	RAMASDFNL	0.3721 <= SB	-0.18	QPDQSESEL	0.3872 <= SB	-1.69
KYTAFTIPSI	8.7662	-1.86	KTGKYARMR<br>KTGKYARMK<br>KTRKYARMR	1.2996 <= WB<br>0.2182 <= SB<br>1.01<= WB	-1.49<br>-0.86<br>-1.01	GADRQGTVSF<br>RADRQGTVSF	1.0843 <= WB<br>0.2516 <= SB	-1.26<br>-0.2	IPAETGQETAY<br>IPAETRQETAY	0.0389 <= SB<br>0.0588 <= SB	0.74<br>0.64
			KVYLAWVPAHK<br>KVYLA*VPAHK	0.2525 <= SB	-0.18	KMIGGIGGF<br>KMIGRIRGF<br>KMIGGIRGF<br>KMIGRIGGF<br>KMIKGIGGF<br>KMIEGIGGF<br>KMIRGIGGF<br>KMIGGIGSF	0.9903 <= WB<br>0.4427 <= SB<br>0.3665 <= SB<br>1.1429 <= WB<br>0.9541<= WB<br>0.8987<= WB<br>1.2474<= WB<br>0.6265<= WB	-1.54<br>-1.22<br>-1.29<br>-1.58<br>-1.61<br>-1.58<br>-1.48<br>-1.28	KAIGTVLV<br>KAISTVLV	18.2581<br>21.506	-4.49<br>-4.48
			KLAGRWPVK<br>KLARRWPVK	0.0249 <= SB<br>0.0772 <= SB	0.2<br>0.3	GGFIKVRQY<br>RGFIKVRQY<br>GSFIKVRQY	1.2180 <= WB<br>0.3642 <= SB<br>0.1954 <= SB	-1.62<br>-0.71<br>-0.52	EKEGKISKI<br>EKEEKISKI<br>EKERKISKI	27.0625<br>25.5854<br>47	-3.46<br>-3.53<br>-3.50
			KIQNFRVYYR<br>KIQNFQVYYK<br>KIQNFRVYYK<br>KIQNFQVYYR	0.9503 <= WB<br>0.1292 <= SB<br>0.1521 <= SB<br>0.8749 <= WB	-0.46<br>-0.13<br>-0.07<br>-0.56	KYTAFTIPSI	1.9718 <= WB	-0.83			
			KVVPRRKAK	0.2018 <= SB		RETKLGKAGY<br>RETKLGKARY<br>RETKLRKAGY<br>KETKLGKAGY<br>RETKLRKRGY	0.8706 <= WB<br>0.5154 <= WB<br>2.1881<= WB<br>0.8961<= WB<br>1.5839<= WB	-0.98<br>-0.72<br>-1.19<br>-1.09<br>-1			
			AVFIHNFKRK	0.2329 <= SB	-0.05	ATVRAACWW<br>ATVKAACWW<br>ATVRAACW*	0.2323 <= SB<br>0.1425 <= SB	0.65<br>0.54			
			IIATDIQTK	0.2684 <= SB	-1.45	FLLKLAGRW<br>FLLKLARRW	1.4404 <= WB<br>0.4775 <= SB	-1.77<br>-1.41			
			KLKPGMDGPK<br>KLKPRIDGPK<br>KLKPRMDGPK<br>KLKPGIDGPK	0.1734 <= SB<br>0.2524 <= SB<br>0.4514 <= SB<br>0.124 <= SB	-1.39<br>-1.32	TVLDVGDAY<br>TVLDVSDAY	1.9631 <= WB<br>1.9631 <= WB	-1.64<br>-1.58			
			GIKVRQLCK<br>RIKVRQLCK<br>EIKVRQLCK	0.8213 <= WB<br>0.4146 <= SB<br>4.6	-1.42<br>-0.62<br>-2.75	GQVDCSPGIW<br>RQVDCSPRIW<br>RQVDCSPGIW<br>GQVDCSPRIW	3.2370<br>0.5925 <= WB<br>1.2071 <= WB<br>1.7597 <= WB	-1.77<br>-0.76<br>-0.90<br>-1.63			
			GVYYDPSK<br>RVYYDPSK	1.2862 <= WB<br>0.4036 <= SB	-2.2<br>-1.31	AETGQETAYF<br>AETRQETAYF	2.24<br>0.5635 <= WB	-1.62<br>-1.2			
			GSNFTGATVR<br>GSNFTGATVK<br>GSNFTSATVR	1.6301 <= WB<br>0.3349 <= SB<br>2.169	-1.29<br>-0.74<br>-1.23						
			QMAVFIHNFK	1.2985 <= WB	-0.27						
			NTPVFAIKK	1.1908 <= WB	-2.23						
			ALVEICTEMEK	2.4074	-1.91						
			SMTKILEPF	21.7308	-0.96						
			KAIGTVLVG<br>KAIGTVLVR	19.0531<br>0.5722 <= WB	-3.91<br>-1.31						
		Gag									
HLA-A2:01			HLA-A3:01			HLA-B57:01			HLA-B35:01		
**Peptide**	**%Rank<=BindLevel ♦**	**Total Score§**	**Peptide**	**%Rank<=BindLevel ♦**	**Total Score§**	**Peptide**	**%Rank<=BindLevel ♦**	**Total Score§**	**Peptide**	**%Rank<=BindLevel ♦**	**Total Score§**
VLAEAMSQV	0.0150 <= SB	0.36	KIRLRPGGKK<br>KIRLRPRGKK<br>KIRLRPEGKK<br>KIRLRPKGKK<br>KIRLRPGRKK<br>KIQLRPGGKK	0.1604 <= SB<br>0.5069 <= WB<br>0.4945 <= SB<br>0.5436 <= WB<br>0.2663 <= SB<br>0.1530 <= SB	-0.82<br>-0.68<br>-1.11<br>-1.05<br>-0.54<br>-1.77	TSTLQEQIGW<br>TSTLQEQIRW<br>TSTLQEQIG*	0.2166 <= SB<br>0.0909 <= SB	0.1<br>0.23	NNPPIPVGEIY<br>NNPPIPVREIY	0.1773 <= SB<br>0.4525 <= SB	0.31<br>0.18
SLYNTVATL	0.1904 <= SB	-0.15	SLYNTVATLY	0.1607 <= SB	1.24	QASQEVKNW<br>QASQEVKN*	0.0552 <= SB	-0.42<br>-0.48	GPAATLEEM<br>RPAATLEEM	0.1176 <= SB<br>0.1825 <= SB	-1.08<br>-0.7
ILGQLQPSL<br>LRQLQPSL	0.2117 <= SB<br>0.91<= WB	-0.18<br>-0.97	KELYPLTSLR<br>KELYPLTSLK	0.1924 <= SB<br>0.0214 <= SB	-0.03<br>0.19	QAISPRTLNAW<br>QAISPRTLNA*	0.2727 <= SB	-0.44	HSNQVSQNY	0.2372 <= SB	0.34
RVLAEAMSQV	0.4484 <= SB	-0.46	KIWPSYKGR<br>KIWPSYKRR<br>KIWPSYKER	0.2742 <= SB<br>0.2498 <= SB<br>0.3007 <= SB	-0.84<br>-0.74	RQANFLGKIW<br>RQANFLRKIW<br>RQANFLEKIW	0.2162 <= SB<br>0.4273 <= SB<br>0.3792 <= SB	0.12<br>-0.04<br>0.1	TPQDLNTML	0.3005 <= SB	-1.05
SLQTGSEEL<br>SLQTRSEEL	0.5473 <= WB<br>1.26<= WB	-0.44<br>-1.01	HQAAMQMLK	0.2857 <= SB	-0.82	KALGPAATL<br>KALRPAATL	0.1618 <= SB<br>0.1723 <= SB	-0.53<br>-0.4	HPVHAGPIA<br>HPVHARPIA	0.4260 <= SB<br>0.9331 <= WB	-1.38<br>-1.92

**MTNNPPIPV**	**1.5397 <= WB**	**-1.87**	**SILDIRQGPK**<br>SILDIRQRPK	**0.3388 <= SB** 0.2920 <= SB	**-1.49**<br>-1.47	**SVLSGGELDRW**<br>SV LSRGELDRW<br>SVLSEGELDRW<br>SVLSKGELDRW<br>SVLSGRELDRW<br>SVLSGGELDR*	**0.4224 <= SB**<br>0.3366 <= SB<br>0.6002 <= WB<br>0.4587 <= SB<br>0.4537 <= SB	**-0.84**<br>-0.73<br>-0.93<br>-.94<br>-0.89	**EPFRDYVDRF**<br>EPFRDYVDQF	**0.4573 <= SB**<br>0.3537 <= SB	**-0.91**<br>-0.73
**TLNAWVKVV**<br>TLNA*VKVV	**1.1521 <= WB**	**-1.52**	**GLNKIVRMY**<br>RLNKIVRMY	**0.3433 <= SB**<br>0.0935 <= SB	**-0.49**<br>0.54	**HSNQVSQNY**	**0.2432 <= SB**	**-0.77**	**GPKEPFRDY**<br>RPKEPFRDY	**0.4771 <= SB**<br>0.3843 <= SB	**-1.24**<br>-1.01
**KELYPLTSL**	**7.4614**	**-2.25**	**FLGKIWPSYK**<br>FLRKIWPSYK<br>FLEKIWPSYK<br>FLEKIWPSYK	**0.5353 <= WB**<br>0.3886 <= SB<br>0.97820 <= WB<br>0.97820 <= WB	**-0.79**<br>-0.46<br>-0.9<br>-0.89	**TINEEAAEW**<br>TINEEAAE*	**0.3099 <= SB**	**-.1.86**	**YPLTSLRSLF**<br>YPLTSLKSLF	**0.5109 <= WB**<br>0.4969 <= SB	**0.52**<br>0.42
**GPGHKARVL**<br>RPGHKARVL	**46.6250**<br>39	**-2.75**<br>-2.65	**GVETTTPPQK**<br>RVETTTPPQK<br>KVETTTPPQK<br>EVETTTPPQK	**0.8126 <= WB**<br>0.2653 <= SB<br>0.2985 <= SB<br>5.0497	**-1.82**<br>-1.17<br>-1.2<br>-3	**KAFSPEVIPMF**	**0.5400 <= WB**	**0.5**	**WASRELERF**<br>WASKELERF<br>*ASRELERF	**1.3704 <= WB**<br>0.6922 <= WB	**-0.45**<br>-0.33
**EMMTACQGV**<br>EMMTACQRV	**3.66**<br>4.66	**-1.07**<br>-1.50	**KLKHIVWASR**<br>KLKHIVWASK<br>KLKHIV*ASR	**1.2530 <= WB**<br>0.1954 <= SB	**-1**<br>-0.33	**FLGKIWPSYK**<br>FLRKIWPSYK<br>FLEKIWPSYK	**0.5353 <= WB**<br>0.3886 <= SB<br>0.9782 <= WB	**-1.75**<br>-1.61<br>-1.85	**EEKAFSPEV**	**9.7969**	**-3.39**
**KRWIILGLNK**<br>KR*IILGLNK	**40.42**	**-3.19**	**ATLYCVHQR**<br>ATLYCVHQK	**1.7586 <= WB**<br>0.3601 <= SB	**-1.47**<br>-0.86	**RAPRKKGCW**<br>KAPRKKGCW<br>RAPKKKGCW<br>RAPRKKGC*	**0.5400 <= WB**<br>0.5877 <= WB<br>0.3867 <= SB	**-1.37**<br>-1.43<br>-1.54			
**EPFRDYVDRF**	**34.03**	**-2.29**	**CVHQRIEIK**<br>CVHQKIEIK	**2.0147**<br>1.9041 <= WB	**-1.8**<br>-2	**IPVGEIYKRW**<br>IPVREIYKRW<br>IPVGEIYKR*	**0.5949 <= WB**<br>0.4140 <= SB	**-1.02**<br>-0.94			
**FLGKIWPSYK**<br>FLRKIWPSYK	**18.49**<br>8.2031	**-3.11**<br>-3.22	**GARASVLSG**<br>GARASVLSK<br>GARASVLSR	**2.5342**<br>0.1501 <= SB	**-4.12**<br>-1.39<br>-1	**GSEELRSLY**<br>RSEELRSLY	**1.2030 <= WB**<br>0.2441 <= SB	**-1.49**<br>-0.6			
**TSTLQEQIGW**<br>TTSTLQEQIRW<br>TSTLQEQIG*	**47.75**<br>60	**-2.83**<br>-2.76	**TIMMQRGNFR**<br>TIMMQRGNF	**2.85**<br>0.5148 <= WB	**-1.1**<br>-0.15	**GLNKIVRMY**<br>RLNKIVRMY	**1.6887 <= WB**<br>0.4303 <= SB	**-1.76**<br>-1.09			
					**Nef**						
**HLA-A2:01**			**HLA-A3:01**			**HLA-B57:01**			**HLA-B35:01**		
**Peptide**	**%Rank<=BindLevel** ♦	**Total Score**§	**Peptide**	**%Rank<=BindLevel** ♦	**Total Score**§	**Peptide**	**%Rank<=BindLevel** ♦	**Total Score**§	**Peptide**	**%Rank<=BindLevel** ♦	**Total Score**§
**GMDDPEREV**<br>RMDDPEREV<br>EIDDPEREV<br>EMDDPEREV<br>GIDDPEREV	**0.3135 <= SB**<br>0.3209 <= SB<br>5.92<br>2.7<br>1.27<= WB	**-1.80**<br>-1.5<br>-3.23<br>-2.76<br>-2.75	**RLAFHHVAR**	**0.1253 <= SB**	**-0.18**	**KWSKSSVIGW**<br>KWSKSSVIRW<br>K*SKSSVIGW	**0.0522 <= SB**<br>0.0252 <= SB	**0.34**<br>0.43	**FPVTPQVPL**	**0.0765 <= SB**	**0.64**
**ILDLWIYHT**<br>ILDL*IYHT	**0.76 <= SB**	**-1.03**	**QVPLRPMTYK**	**0.2292 <= SB**	**-0.56**	**KAAVDLSHF**	**0.1157 <= SB**	**0.15**	**YPLTFGWCY**<br>YPLTFRWCY<br>YPLTFG*CY	**0.0949 <= SB**<br>0.1733<= SB	**1.76**<br>1.68
**FLKEKGGLEGL**<br>FLKEKGGLERL<br>FLKEKKGLERL<br>FLKEKKGLEGL<br>FLKEKGRLERL<br>FLKEKGRLEGL	**1.302 <= WB**<br>1.4072<= WB<br>1.4085<= WB<br>1.3046<= WB<br>4.2<br>4.7	**-1.25**<br>-1.37<br>-1.37<br>-1.32<br>-1.67<br>-1.78	**KLVPVEPDK**	**0.2297 <= SB**	**-1.81**	**VARELHPEY**	**0.2500 <= SB**	**-0.81**	**VARELHPEY**	**0.0980 <= SB**	**0.72**
**KLVPVEPDKI**	**0.9915 <= WB**	**-1.59**	**SVIGWPTVR**<br>SVIGWPTVK<br>SVIRWPTVR	**0.3479 <= SB**<br>0.021<= SB<br>0.446 <= SB	**-0.50**<br>-1.02<br>-1.15	**AATNAACAW**	**0.2689 <= SB**	**-0.16**	**QVPLRPMTY**	**0.7614 <= SB**	**-0.79**
**VLEWRFDSRL**<br>VLE*RFDSRL	**3.43**	**-0.99**	**AAVDLSHFLK**	**0.7043 <= WB**	**-1.23**	**GVRYPLTFGW**<br>RVRYPLTFGW<br>KVRYPLTFGW<br>GVRYPLTFRW<br>GVRYPLTFG*	**0.4492 <= SB**<br>0.1060 <= SB<br>0.1140 <= SB<br>0.2428 <= SB	**-0.22**<br>0.62<br>0.63<br>-0.02	**YTPGPGVRY**<br>YTPRPRVRY<br>YTPRPKVRY<br>YTPRPGVRY<br>YTPGPKVRY<br>YTPGPRVRY	**0.4460 <= SB**<br>1.2403 <= WB<br>1.419 <= WB<br>0.818 <= WB<br>0.698 <= WB<br>0.5927 <= WB	**-0.81**<br>-1.13<br>-1.28<br>-0.99<br>-1.08<br>-0.90
**QVPLRPMTYK**	**41.916**	**-3.54**	**RVGAASRDLEK**<br>KVGAASRDLEK<br>RVRAASRDLEK	**1.0908 <= WB**<br>1.2183 <= WB<br>0.8476 <= WB	**-1.85**<br>-1.90<br>-1.35	**YTPGPGVRY**<br>YTPRPRVRY<br>YTPRPKVRY<br>YTPRPGVRY<br>YTPGPKVRY<br>YTPGPRVRY	**0.7305 <= WB**<br>0.5308 <= WB<br>0.5709 <= WB<br>0.6417 <= WB<br>0.6712 <= WB<br>0.619 <= WB	**-1.40**<br>-1.43<br>-1.43<br>-1.41<br>-1.45<br>-1.42	**HPVSLHGM**<br>HPVSLHRM<br>HPVSLHEI<br>HPVSLHGI<br>HPVSLHEM	**0.9238 <= WB**<br>0.8613 <= WB<br>2.73<br>5.1<br>0.3755<= **SB**	**-2.56**<br>-2.63<br>-2.77<br>-2.95<br>-1.99
**PLTFGWCYKL**<br>PLTFG*CYKL	**19.4560**	**-1.45**	**SLLHPVSLHG**<br>SLLHPVSLHR	**7.1854**<br>0.2899 <= SB	**-3.53**<br>-0.34	**VGFPVTPQV**<br>VSFPVTPQV	**1.4842 <= WB**<br>0.2993 <= SB	**-2.56**<br>-1.53	**YFPDWQNYT**<br>YFPD*QNYT	**18.76**	**-3.91**

AFHHVAREL	11.9	-2.36	RMRRAEPAADR<br>RMRRAEPAADK	45.6000<br>0.8247 <= WB	-1.84<br>-1.40	HTQGYFPDWQ<br>HTQGYFPD*Q	3.90	-2.56	FLKEKGGL<br>FLKEKGRL<br>FLKEKRKL<br>FLKEKRGL<br>FLKEKKGL	32.5294<br>28<br>32<br>36<br>40	-2.98<br>-2.92<br>-2.85<br>-2.89<br>-2.81
			GVRYPLTFG<br>GVRYPLTFR	37.6111<br>0.1529 <= SB	-3.98<br>-0.52	YFPDWQNYT<br>YFPD*QNYT	41.1724	-4.05			
Env											
HLA-A2:01			HLA-A3:01			HLA-B57:01			HLA-B35:01		
Peptide	%Rank<=BindLevel [♦]	Total Score[§]	Peptide	%Rank<=BindLevel [♦]	Total Score[§]	Peptide	%Rank<=BindLevel [♦]	Total Score[§]	Peptide	%Rank<=BindLevel [♦]	Total Score[§]
QMHEDIISL	0.0767 <= SB	0.45	KLREQFGNNK<br>KLREQFRNNK	0.0786 <= SB<br>0.1807 <= SB	-0.48<br>-0.59	STQLFNSTW<br>STQLFNST*	0.0094 <= SB	0.67	IPIDNDTTSY	0.0115 <= SB	1.49
KLTPLCVSL	0.3144 <= SB	0.57	TVYYGVPVWK<br>TVYYRVPVWK<br>TVYYKVPVWK<br>TVYYEVPVWK<br>TVYYGVPV*K	0.1141 <= SB<br>0.3323 <= SB<br>0.2281 <= SB<br>0.2861 <= SB	-0.17<br>-0.11<br>-0.27<br>-0.43	RIKQIINMW	0.0167 <= SB	-0.2	DPEIVTHSF	0.0944 <= SB	-0.21
RLVNGSLAL<br>RLVNRSLAL	0.1790 <= SB<br>0.4885 <= SB	0.10<br>-0.31	KLTPLCVSLK	0.1937 <= SB	-0.21	TVYYGVPVW<br>TVYYRVPVW<br>TVYYKVPVW<br>TVYYEVPVW<br>TVYYGVPV*	0.0346 <= SB<br>0.1140 <= SB<br>0.0879 <= SB<br>0.0508 <= SB	0.1<br>-0.01<br>-0.08<br>0.14	DPNPQEVVL	0.2066 <= SB	-0.72
SLAEEEVVI	0.1682 <= SB	-0.6	SELYKYKVVK	0.2867 <= SB	-0.37	KAYDTEVHNVW<br>KAYDTEVHNV*	0.0397 <= SB	0.82	SPLSFQTHL	0.5053 <= WB	-1.4
ALIWDDLRSL<br>ALIWDDLQSL<br>ALI*DDLRSL	0.2266 <= SB<br>0.071 4 <= SB	0.06<br>0.58	SSGRMIMEK<br>SSRRMIMEK<br>SSERMIIEK<br>SSERMIMEK<br>SSGRMIIEK	0.2586 <= SB<br>0.2586 <= SB<br>1.0651 <= WB<br>0.8763 <= WB<br>0.3909 <= SB	-1.23<br>-0.8<br>-1.98<br>-1.77<br>-1.44	NSTWFNSTW<br>NST*FNST*	0.1373 <= SB	0.39	FCASDAKAY	0.3328 <= SB	1.15
AVLSIVNRV	0.2642 <= SB	-0.79	NTLKQIASK	0.3169 <= SB	-1.79	IASKLREQF	0.1434 <= SB	-0.13	VPVWKEATTTL<br>VPV*KEATTTL	1.8948 <= WB	-1.94
SLLNATAIAV	0.3428 <= SB	0.01	STSIRGKVQK<br>STSIRSKVQK	0.3792 <= SB<br>0.5362 <= WB	-1.16<br>-1.31	VSFEPIPIHY	0.2367 <= SB	0.37	RAVGIGALF<br>RAVRIRALF<br>RAVGIRALF<br>RAVRIGALF	0.2928 <= SB<br>1.6833 <= WB<br>0.5752 <= WB<br>0.7789 <= WB	-0.01<br>-1.07<br>-0.47<br>-0.64
ALFLGFLGA<br>ALFLRFLGA<br>ALFLGFLRA<br>ALFLEFLGA	0.2868 <= SB<br>0.7989 <= WB<br>0.2560 <= SB<br>0.3879 <= SB	-0.45<br>-0.67<br>-0.68<br>-0.53	RSVNFTDNAK	0.3797 <= SB	-0.7	ICSATEKLW<br>ICSATEKL*	0.2718 <= SB	-0.47	TAVPWNASW<br>TAVPWNAS*<br>TAVP*NASW	0.2964 <= SB	-0.78
IMIVGGLVGL<br>IMIVRGLVGL<br>IMIVGGLVSL	0.3766 <= SB<br>1.6154 <= WB<br>0.3361 <= SB	0.68<br>0.29<br>0.7	RAKWNNTLK<br>RAK*NNTLK	0.4303 <= SB	-1.23	HCNISRAKW<br>HCNISRAK*	0.2925 <= SB	-1.06			
AVAEGTDRV<br>AVAEGTDKV<br>AVAERTDKV<br>AVAERTDRV<br>AVAEETDRV	0.4306 <= SB<br>0.4358 <= SB<br>0.7732 <= WB<br>0.8261 <= WB<br>0.6285 <= WB	-1.32<br>-1.44<br>-1.95<br>-1.88<br>-1.76	KQIINMWQK	0.4955 <= SB	-1.22	NVTENFNMW<br>NVTENFNM*	0.3244 <= SB	-1.25			
RLRDLLLIV	0.5094 <= WB	-0.47	VTENFNMWK<br>VTENFNM*K	0.7215 <= WB	-1.03	KVQKEYAFF	0.3400 <= SB	-0.6			
QLQARILAV	0.5520 <= WB	-1	RIVELLGRR<br>RIVELLGRK<br>KIVELLRRR<br>RIVELLRRR<br>KIVELLGRR<br>RIVELLERR	0.6685 <= WB<br>0.0585 <= SB<br>0.7459 <= WB<br>0.6796 <= WB<br>0.7433 <= WB<br>1.19<= WB	-1.44<br>-0.81<br>-1.49<br>-1.45<br>-1.49<br>-1.84	HLWRWGWRW<br>HLWRWRWRW<br>HLWRWEWRW<br>HLWRWGWR*<br>HL*RWGWRW<br>HLWR*GWRW<br>HLWRWG*RW	0.5696 <= WB<br>0.8413 <= WB<br>0.8565 <= WB	-0.67<br>-0.45<br>-0.37			
FLGAAGSTM<br>FLGAAGSTI<br>FLRAARSTM<br>FLRAAGSTM<br>FLGAARSTM	0.6744 <= WB<br>0.3906 <= SB<br>6.4642<br>4.9805<br>1.6432 <= WB	-1.36<br>-0.58<br>-2.46<br>-2.06<br>-1.79	RMIMEKGEIK<br>RMIIEKREIK<br>RMIIEKGEIK<br>RMIMEKREIK	0.3398 <= SB<br>0.8917 <= WB<br>0.5269 <= WB<br>0.669 <= WB	-0.73<br>-1.16<br>-1.06<br>-0.82	RAFVTIGKI<br>RAFVTIRKI	1.0234 <= WB<br>0.3638 <= SB	-1.38<br>-1.08			
KLTSCNTS	0.6547 <= WB	-0.49	RILAVERYLK	0.3652 <= SB	-0.94	KRAVGIGALF<br>KRAVRIRALF<br>KRAVGIRALF<br>KRAVRIGALF	0.2677 <= SB<br>0.7974 <= WB<br>0.4617 <= SB<br>0.4546 <= SB	0.14<br>0.06<br>0.02<br>0.21			
RVIEVVQGA<br>KVIEVVQGA<br>RVIEVVQRA	0.7116 <= WB<br>0.5425 <= WB<br>0.9374 <= WB	-1.6<br>-1.45<br>-2.06	RYLKDQQLLG<br>RYLKDQQLLR	22.0000<br>0.5276 <= WB	-3.97<br>-1	RGWEALKYW<br>KGWEALKYW<br>KRWEALKYW<br>RRWEALKYW	0.0334 <= SB<br>0.0364 <= SB<br>0.7474 <= WB<br>0.7742 <= WB	0.33<br>0.32<br>-1.45<br>-1.58			

						REWEALKYW<br>RG*EALKYW<br>RGWEALKY*	0.6480 <= WB	-1.51		
**YLKDQQLLGI**<br>YLKDQQLLRI<br>YLKDQQLLEI	**0.7800 <= WB**<br>0.7800 <= WB<br>0.5065 <= WB	**-0.67**<br>-1	**IIFKQSSGG**<br>IIFKQSSGR<br>IIFKQSSGK	**20.3846**<br>0.2925 <= SB<br>0.0225 <= SB	**-4.27**<br>-0.77<br>-0.18	**TTAVPWNASW**<br>TTAVP*NASW<br>TTAVPWNAS*	**0.0539 <= SB**	**0.44**		
**FIMIVGGLVGL**<br>FIMIVGGLVSL<br>FIMIVRGLVGL	**0.8180 <= WB**<br>0.7468 <= WB<br>3.4482	**0.26**<br>0.33<br>-0.24	**KLWVTVYYG**<br>KLWVTVYYR<br>KLWVTVYYK<br>KL*VTVYYG	**13.5977**<br>0.5323 <= WB<br>0.1399 <= SB	**-3.86**<br>-0.55<br>-0.22	**RAIEAQQHL**<br>KAIEAQQHL	**0.1037 <= SB**<br>0.0977 <= SB	**-0.58**<br>-0.68		
**LLQYWSQEL**<br>LLQY*SQEL	**1.0574 <= WB**	**0.37**				**WSNKSLEQIW**<br>*SNKSLEQIW<br>WSNKSLEQI*	**0.5692 <= WB**	**0.31**		
**IISLWDQSL**<br>IISL*DQSL	**2.6161**	**-0.96**				**VNGSLALIW**<br>VNRSLALIW<br>VNGSLALI*	**1.2356 <= WB**<br>0.4569 <= SB	**0.89**<br>0.92		
**NVWATHACV**<br>NV*ATHACV	**2.589**	**-0.78**								
**RGPGRAFVTI**<br>RRPERAFVTI<br>RRPEGAFVTI<br>RGPERAFVTI	**21.8931**<br>20.49<br>20.06<br>16.30	**-2.75**<br>-2.72<br>-2.72<br>-2.76								
**IPRRIRQGL**	**32.862**	**-2.83**								

♦ **%Rank** Rank of the predicted affinity compared to a set of random natural peptides. This measure is not affected by inherent bias of certain molecules towards higher or lower mean predicted affinities. Strong binders are defined as having %rank<0.5, and weak binders with %rank<2 (Jurtz et al., 2017) (http://www.cbs.dtu.dk/services/NetMHCpan/).

♦ **BindLevel** (SB: strong binder, WB: weak binder). The peptide will be identified as a strong binder if the % Rank is below the specified threshold for the strong binders, by default 0.5%. The peptide will be identified as a weak binder if the % Rank is above the threshold of the strong binders but below the specified threshold for the weak binders, by default 2% (Jurtz et al., 2017) (http://www.cbs.dtu.dk/services/NetMHCpan/).

§ **Total score** This score combines the proteasomal cleavage, TAP transport and MHC binding predictions. It predicts a quantity proportional to amount of peptide presented by MHC molecules on the cell surface (http://tools.iedb.org/processing).

Predicted wild type epitopes are black bolded (http://www.cbs.dtu.dk/services/NetMHCpan/)
Known wild type epitopes are red bolded (https://www.hiv.lanl.gov/content/immunology/tables/ctl_summary.html)
GGG hotspot motif altered peptides and * stop codon are green bolded
GGA hotspot motif altered peptides and * stop codon are orange bolded
GGT hotspot motif altered peptides and * stop codon are blue bolded
Novel predicted epitopes are in orange cells

Table S2: Enrichment of A3G hotspot motifs (GGG, GGA and GGT) inside/outside HLA-A2, HLA-A3, HLA-B57 and HLA-B35-restricted CTL epitopes for Pol, Gag, Nef and Env using NetMHCpan 4 and known CTL epitopes using traditional experimental discovery methods viral genomic nucleotide and protein sequence levels

Pol

Hotspot / HLA	HLA-A3:01			
GGA	motif			
GGA	85			
GGA	Normalized Motif Inside CTL-epitopes		Normalized Motif Outside CTL-epitopes	
GGA	0.021		0.031	
GGA	Motif In/out CTL-epitopes<br>0.67			
GGA	Normalized Inside CTL-epitopes		Normalized Outside CTL-epitopes	
GGA	Nonsynonymous mutations (including stop codon)	Silent mutations	Nonsynonymous mutations (including stop codon)	Silent mutations
GGA	0.048	0.013	0.075	0.018
GGA	Ratio of all instances In/outside CTL-epitopes			
GGA	0.66			
GGA	Ratio of nonsynonymous to silent mutations In/outside CTL-epitopes			
GGA	0.88			
GGA	Ratio of nonsynonymous mutations In/outside CTL-epitopes		Ratio of silent mutations In/outside CTL-epitopes	
GGA	0.64		0.72	
GGG	motif			
GGG	58			
GGG	Normalized Motif Inside CTL-epitopes		Normalized Motif Outside CTL-epitopes	
GGG	0.019		0.031	
GGG	Motif In/out CTL-epitopes<br>0.61			
GGG	Normalized Inside CTL-epitopes		Normalized Outside CTL-epitopes	
GGG	Nonsynonymous mutations (including stop codon)	Silent mutations	Nonsynonymous mutations (including stop codon)	Silent mutations
GGG	0.051	0	0.050	0.0084
GGG	Ratio of all instances In/outside CTL-epitopes			
GGG	0.86			
GGG	Ratio of nonsynonymous to silent mutations In/outside CTL-epitopes			
GGG	2.4			
GGG	Ratio of nonsynonymous mutations In/outside CTL-epitopes		Ratio of silent mutations In/outside CTL-epitopes	
GGG	1.02		0	
GGT	motif			
GGT	24			
GGT	Normalized Motif Inside CTL-epitopes		Normalized Motif Outside CTL-epitopes	
GGT	0.0091		0.00748	
GGT	Motif In/out CTL-epitopes<br>1.21			

Hotspot / HLA	HLA-A2:01			
GGA	motif			
GGA	85			
GGA	Normalized Motif Inside CTL-epitopes		Normalized Motif Outside CTL-epitopes	
GGA	0.0313		0.0274	
GGA	Motif In/out CTL-epitopes<br>1.148			
GGA	Normalized Inside CTL-epitopes		Normalized Outside CTL-epitopes	
GGA	Nonsynonymous mutations (including stop codon)	Silent mutations	Nonsynonymous mutations (including stop codon)	Silent mutations
GGA	0.094	0.01	0.061	0.018
GGA	Ratio of all instances In/outside CTL-epitopes			
GGA	1.25			
GGA	Ratio of nonsynonymous to silent mutations In/outside CTL-epitopes			
GGA	2.76			
GGA	Ratio of nonsynonymous mutations In/outside CTL-epitopes		Ratio of silent mutations In/outside CTL-epitopes	
GGA	1.54		0.55	
GGG	motif			
GGG	58			
GGG	Normalized Motif Inside CTL-epitopes		Normalized Motif Outside CTL-epitopes	
GGG	0.016		0.0199	
GGG	Motif In/out CTL-epitopes<br>0.8			
GGG	Normalized Inside CTL-epitopes		Normalized Outside CTL-epitopes	
GGG	Nonsynonymous mutations (including stop codon)	Silent mutations	Nonsynonymous mutations (including stop codon)	Silent mutations
GGG	0.044	0.01	0.052	0.005
GGG	Ratio of all instances In/outside CTL-epitopes			
GGG	0.94			
GGG	Ratio of nonsynonymous to silent mutations In/outside CTL-epitopes			
GGG	0.42			
GGG	Ratio of nonsynonymous mutations In/outside CTL-epitopes		Ratio of silent mutations In/outside CTL-epitopes	
GGG	0.84		2	
GGT	motif			
GGT	24			
GGT	Normalized Motif Inside CTL-epitopes		Normalized Motif Outside CTL-epitopes	
GGT	0.0082		0.008	
GGT	Motif In/out CTL-epitopes<br>1.025			

Hotspot / HLA	HLA-B57:01			
GGA	motif			
GGA	85			
GGA	Normalized Motif Inside CTL-epitopes		Normalized Motif Outside CTL-epitopes	
GGA	0.031		0.027	
GGA	Motif In/out CTL-epitopes<br>1.14			
GGA	Normalized Inside CTL-epitopes		Normalized Outside CTL-epitopes	
GGA	Nonsynonymous mutations (including stop codon)	Silent mutations	Nonsynonymous mutations (including stop codon)	Silent mutations
GGA	0.071	0.015	0.066	0.0175
GGA	Ratio of all instances In/outside CTL-epitopes			
GGA	1.048			
GGA	Ratio of nonsynonymous to silent mutations In/outside CTL-epitopes			
GGA	1.25			
GGA	Ratio of nonsynonymous mutations In/outside CTL-epitopes		Ratio of silent mutations In/outside CTL-epitopes	
GGA	1.075		0.85	
GGG	motif			
GGG	58			
GGG	Normalized Motif Inside CTL-epitopes		Normalized Motif Outside CTL-epitopes	
GGG	0.025		0.017	
GGG	Motif In/out CTL-epitopes<br>1.47			
GGG	Normalized Inside CTL-epitopes		Normalized Outside CTL-epitopes	
GGG	Nonsynonymous mutations (including stop codon)	Silent mutations	Nonsynonymous mutations (including stop codon)	Silent mutations
GGG	0.034	0.0075	0.056	0.0054
GGG	Ratio of all instances In/outside CTL-epitopes			
GGG	0.66			
GGG	Ratio of nonsynonymous to silent mutations In/outside CTL-epitopes			
GGG	0.437			
GGG	Ratio of nonsynonymous mutations In/outside CTL-epitopes		Ratio of silent mutations In/outside CTL-epitopes	
GGG	0.6		1.39	
GGT	motif			
GGT	24			
GGT	Normalized Motif Inside CTL-epitopes		Normalized Motif Outside CTL-epitopes	
GGT	0.0138		0.0058	
GGT	Motif In/out CTL-epitopes<br>2.37			

Hotspot / HLA	HLA-B35:01			
GGA	motif			
GGA	85			
GGA	Normalized Motif Inside CTL-epitopes		Normalized Motif Outside CTL-epitopes	
GGA	0.029		0.027	
GGA	Motif In/out CTL-epitopes<br>1.07			
GGA	Normalized Inside CTL-epitopes		Normalized Outside CTL-epitopes	
GGA	Nonsynonymous mutations (including stop codon)	Silent mutations	Nonsynonymous mutations (including stop codon)	Silent mutations
GGA	0.059	0.02	0.0699	0.0162
GGA	Ratio of all instances In/outside CTL-epitopes			
GGA	0.918			
GGA	Ratio of nonsynonymous to silent mutations In/outside CTL-epitopes			
GGA	0.68			
GGA	Ratio of nonsynonymous mutations In/outside CTL-epitopes		Ratio of silent mutations In/outside CTL-epitopes	
GGA	0.84		1.234	
GGG	motif			
GGG	58			
GGG	Normalized Motif Inside CTL-epitopes		Normalized Motif Outside CTL-epitopes	
GGG	0.01		0.021	
GGG	Motif In/out CTL-epitopes<br>0.476			
GGG	Normalized Inside CTL-epitopes		Normalized Outside CTL-epitopes	
GGG	Nonsynonymous mutations (including stop codon)	Silent mutations	Nonsynonymous mutations (including stop codon)	Silent mutations
GGG	0.024	0.005	0.057	0.0062
GGG	Ratio of all instances In/outside CTL-epitopes			
GGG	0.47			
GGG	Ratio of nonsynonymous to silent mutations In/outside CTL-epitopes			
GGG	0.52			
GGG	Ratio of nonsynonymous mutations In/outside CTL-epitopes		Ratio of silent mutations In/outside CTL-epitopes	
GGG	0.42		0.80	
GGT	motif			
GGT	24			
GGT	Normalized Motif Inside CTL-epitopes		Normalized Motif Outside CTL-epitopes	
GGT	0.008		0.0079	
GGT	Motif In/out CTL-epitopes<br>1.012			

	Normalized Inside CTL-epitopes		Normalized Outside CTL-epitopes		Normalized Inside CTL-epitopes		Normalized Outside CTL-epitopes		Normalized Inside CTL-epitopes		Normalized Outside CTL-epitopes		Normalized Inside CTL-epitopes		Normalized Outside CTL-epitopes	
	Normalized Inside CTL-epitopes	Normalized Outside CTL-epitopes	Normalized Inside CTL-epitopes	Normalized Outside CTL-epitopes	Normalized Inside CTL-epitopes	Normalized Outside CTL-epitopes	Normalized Inside CTL-epitopes	Normalized Outside CTL-epitopes	Normalized Inside CTL-epitopes	Normalized Outside CTL-epitopes	Normalized Inside CTL-epitopes	Normalized Outside CTL-epitopes	Normalized Inside CTL-epitopes	Normalized Outside CTL-epitopes	Normalized Inside CTL-epitopes	no aa changes
	0.017	0.010	0.017	0.0056	0.019	0.005	0.016	0.0075	0.03	0.011	0.012	0.0054	0.02	0	0.0162	0.0087
	Ratio of all instances In/outside CTL-epitopes				Ratio of all instances In/outside CTL-epitopes				Ratio of all instances In/outside CTL-epitopes				Ratio of all instances In/outside CTL-epitopes			
	1.23				1.04				2.34				0.8			
	Ratio of nonsynonymous to silent mutations In/outside CTL-epitopes				Ratio of nonsynonymous to silent mutations In/outside CTL-epitopes				Ratio of nonsynonymous to silent mutations In/outside CTL-epitopes				Ratio of nonsynonymous to silent mutations In/outside CTL-epitopes			
	0.56				1.78				1.23				2.15			
	Ratio of nonsynonymous mutations In/outside CTL-epitopes		Ratio of silent mutations In/outside CTL-epitopes		Ratio of nonsynonymous mutations In/outside CTL-epitopes		Ratio of silent mutations In/outside CTL-epitopes		Ratio of nonsynonymous mutations In/outside CTL-epitopes		Ratio of silent mutations In/outside CTL-epitopes		Ratio of nonsynonymous mutations In/outside CTL-epitopes		Ratio of silent mutations In/outside CTL-epitopes	
	1		1.78		1.18		0.66		2.5		2.03		1.23		0	
Gag																
Motif / HLA	**HLA-A3:01**				**HLA-A2:01**				**HLA-B57:01**				**HLA-B35:01**			
GGA	motif				motif				motif				motif			
	42				42				42				42			
	Normalized Motif Inside CTL-epitopes		Normalized Motif Outside CTL-epitopes		Normalized Motif Inside CTL-epitopes		Normalized Motif Outside CTL-epitopes		Normalized Motif Inside CTL-epitopes		Normalized Motif Outside CTL-epitopes		Normalized Motif Inside CTL-epitopes		Normalized Motif Outside CTL-epitopes	
	0.036		0.025		0.032		0.026		0.045		0.022		0.012		0.023	
	Motif In/out CTL-epitopes 1.44				Motif In/out CTL-epitopes 1.23				Motif In/out CTL-epitopes 2				Motif In/out CTL-epitopes 0.52			
	Normalized Inside CTL-epitopes		Normalized Outside CTL-epitopes		Normalized Inside CTL-epitopes		Normalized Outside CTL-epitopes		Normalized Inside CTL-epitopes		Normalized Outside CTL-epitopes		Normalized Inside CTL-epitopes		Normalized Outside CTL-epitopes	
	Nonsynonymous mutations (including stop codon)	Silent mutations	Nonsynonymous mutations (including stop codon)	Silent mutations	Nonsynonymous mutations (including stop codon)	Silent mutations	Nonsynonymous mutations (including stop codon)	Silent mutations	Nonsynonymous mutations (including stop codon)	Silent mutations	Nonsynonymous mutations (including stop codon)	Silent mutations	Nonsynonymous mutations (including stop codon)	Silent mutations	Nonsynonymous mutations (including stop codon)	Silent mutations
	0.13	0.03	0.045	0.02	0.083	0.0138	0.053	0.025	0.11	0.025	0.047	0.0211	0.037	0.009	0.05	0.015
	Ratio of all instances In/outside CTL-epitopes				Ratio of all instances In/outside CTL-epitopes				Ratio of all instances In/outside CTL-epitopes				Ratio of all instances In/outside CTL-epitopes			
	2.46				1.24				2				1			
	Ratio of nonsynonymous to silent mutations In/outside CTL-epitopes				Ratio of nonsynonymous to silent mutations In/outside CTL-epitopes				Ratio of nonsynonymous to silent mutations In/outside CTL-epitopes				Ratio of nonsynonymous to silent mutations In/outside CTL-epitopes			
	1.92				2.81				1.97				0.33			
	Ratio of Nonsynonymous mutations In/outside CTL-epitopes		Ratio of silent mutations In/outside CTL-epitopes		Ratio of Nonsynonymous mutations In/outside CTL-epitopes		Ratio of silent mutations In/outside CTL-epitopes		Ratio of Nonsynonymous mutations In/outside CTL-epitopes		Ratio of silent mutations In/outside CTL-epitopes		Ratio of Nonsynonymous mutations In/outside CTL-epitopes		Ratio of silent mutations In/outside CTL-epitopes	
	2.88		1.5		1.56		0.55		2.12		1.18		0.69		2.07	
GGG	motif				motif				motif				motif			
	32				32				32				32			
	Normalized Motif Inside CTL-epitopes		Normalized Motif Outside CTL-epitopes		Normalized Motif Inside CTL-epitopes		Normalized Motif Outside CTL-epitopes		Normalized Motif Inside CTL-epitopes		Normalized Motif Outside CTL-epitopes		Normalized Motif Inside CTL-epitopes		Normalized Motif Outside CTL-epitopes	
	0.03		0.02		0.02		0.021		0.030		0.018		0.012		0.023	
	Motif In/out CTL-epitopes 1.5				Motif In/out CTL-epitopes 0.95				Motif In/out CTL-epitopes 1. 6				Motif In/out CTL-epitopes 0.52			
	Normalized Inside CTL-epitopes		Normalized Outside CTL-epitopes		Normalized Inside CTL-epitopes		Normalized Outside CTL-epitopes		Normalized Inside CTL-epitopes		Normalized Outside CTL-epitopes		Normalized Inside CTL-epitopes		Normalized Outside CTL-epitopes	
	Nonsynonymous mutations (including stop codon)	Silent mutations	Nonsynonymous mutations (including stop codon)	Silent mutations	Nonsynonymous mutations (including stop codon)	Silent mutations	Nonsynonymous mutations (including stop codon)	Silent mutations	Nonsynonymous mutations (including stop codon)	Silent mutations	Nonsynonymous mutations (including stop codon)	Silent mutations	Nonsynonymous mutations (including stop codon)	Silent mutations	Nonsynonymous mutations (including stop codon)	Silent mutations
	0.05	0.02	0.047	0.01	0.034	0.027	0.050	0.0056	0.057	0.024	0.044	0.0079	0.037	0.009	0.05	0.015
	Ratio of all instances In/outside CTL-epitopes				Ratio of all instances In/outside CTL-epitopes				Ratio of all instances In/outside CTL-epitopes				Ratio of all instances In/outside CTL-epitopes			
	1.22				1.05				1.57				0.56			
	Ratio of nonsynonymous to silent mutations In/outside CTL-epitopes				Ratio of nonsynonymous to silent mutations In/outside CTL-epitopes				Ratio of nonsynonymous to silent mutations In/outside CTL-epitopes				Ratio of nonsynonymous to silent mutations In/outside CTL-epitopes			
	0.52				0.14				0.426				1.24			
	Ratio of Nonsynonymous mutations In/outside CTL-epitopes		Ratio of silent mutations In/outside CTL-epitopes		Ratio of Nonsynonymous mutations In/outside CTL-epitopes		Ratio of silent mutations In/outside CTL-epitopes		Ratio of Nonsynonymous mutations In/outside CTL-epitopes		Ratio of silent mutations In/outside CTL-epitopes		Ratio of Nonsynonymous mutations In/outside CTL-epitopes		Ratio of silent mutations In/outside CTL-epitopes	
	1.01		2		0.68		4.8		1.29		3		0.74		0.6	
GGT	motif				motif				motif				motif			
	11				11				11				11			
	Normalized Motif Inside CTL-epitopes		Normalized Motif Outside CTL-epitopes		Normalized Motif Inside CTL-epitopes		Normalized Motif Outside CTL-epitopes		Normalized Motif Inside CTL-epitopes		Normalized Motif Outside CTL-epitopes		Normalized Motif Inside CTL-epitopes		Normalized Motif Outside CTL-epitopes	
	0.01		0.006		0.0069		0.0075		0.0055		0.008		0.0124		0.006	
	Motif In/out CTL-epitopes 1.6				Motif In/out CTL-epitopes 0.92				Motif In/out CTL-epitopes 0.625				Motif In/out CTL-epitopes 2			
	Normalized Inside CTL-epitopes		Normalized Outside CTL-epitopes		Normalized Inside CTL-epitopes		Normalized Outside CTL-epitopes		Normalized Inside CTL-epitopes		Normalized Outside CTL-epitopes		Normalized Inside CTL-epitopes		Normalized Outside CTL-epitopes	
	Normalized	Normalized	Normalized	Normalized	Normalized	Normalized	Normalized	Normalized	Normalized	Normalized	Normalized	Normalized	Normalized	Normalized	Normalized	no aa changes

	Inside CTL-epitopes	Outside CTL-epitopes	Inside CTL-epitopes	Outside CTL-epitopes	Inside CTL-epitopes	Outside CTL-epitopes	Inside CTL-epitopes	Outside CTL-epitopes	Inside CTL-epitopes	Outside CTL-epitopes	Inside CTL-epitopes	Outside CTL-epitopes	Inside CTL-epitopes	Outside CTL-epitopes	Inside CTL-epitopes	
	0.02	0.01	0.016	0.01	0.02	0.007	0.011	0.011	0.0082	0.0165	0.0158	0.0052	0.019	0.019	0.0128	0.005
	Ratio of all instances In/outside CTL-epitopes				Ratio of all instances In/outside CTL-epitopes				Ratio of all instances In/outside CTL-epitopes				Ratio of all instances In/outside CTL-epitopes			
	1.15				0.1				1.14				2			
	Ratio of nonsynonymous to silent mutations In/outside CTL-epitopes				Ratio of nonsynonymous to silent mutations In/outside CTL-epitopes				Ratio of nonsynonymous to silent mutations In/outside CTL-epitopes				Ratio of nonsynonymous to silent mutations In/outside CTL-epitopes			
	1.25				2.85				0.154				0.4			
	Ratio of Nonsynonymous mutations In/outside CTL-epitopes		Ratio of silent mutations In/outside CTL-epitopes		Ratio of Nonsynonymous mutations In/outside CTL-epitopes		Ratio of silent mutations In/outside CTL-epitopes		Ratio of Nonsynonymous mutations In/outside CTL-epitopes		Ratio of silent mutations In/outside CTL-epitopes		Ratio of Nonsynonymous mutations In/outside CTL-epitopes		Ratio of silent mutations In/outside CTL-epitopes	
	1.25		1		1.81		0		0.52		3.17		1.48		3.8	
Nef																
Motif / HLA	**HLA-A3:01**				**HLA-A2:01**				**HLA-B57:01**				**HLA-B35:01**			
GGA	motif				motif				motif				motif			
	18				18				18				18			
	Normalized Motif Inside CTL-epitopes		Normalized Motif Outside CTL-epitopes		Normalized Motif Inside CTL-epitopes		Normalized Motif Outside CTL-epitopes		Normalized Motif Inside CTL-epitopes		Normalized Motif Outside CTL-epitopes		Normalized Motif Inside CTL-epitopes		Normalized Motif Outside CTL-epitopes	
	0.023		0.031		0.035		0.025		0.014		0.037		0.023		0.031	
	Motif In/out CTL-epitopes				Motif In/out CTL-epitopes				Motif In/out CTL-epitopes				Motif In/out CTL-epitopes			
	0.74				1.4				0.378				0.74			
	Normalized Inside CTL-epitopes		Normalized Outside CTL-epitopes		Normalized Inside CTL-epitopes		Normalized Outside CTL-epitopes		Normalized Inside CTL-epitopes		Normalized Outside CTL-epitopes		Normalized Inside CTL-epitopes		Normalized Outside CTL-epitopes	
	Nonsynonymous mutations (including stop codon)	Silent mutations	Nonsynonymous mutations (including stop codon)	Silent mutations	Nonsynonymous mutations (including stop codon)	Silent mutations	Nonsynonymous mutations (including stop codon)	Silent mutations	Nonsynonymous mutations (including stop codon)	Silent mutations	Nonsynonymous mutations (including stop codon)	Silent mutations	Nonsynonymous mutations (including stop codon)	Silent mutations	Nonsynonymous mutations (including stop codon)	Silent mutations
	0.035	0.0175	0.053	0.046	0.08	0.026	0.030	0.045	0.042	0.014	0.052	0.052	0.071	0.0178	0.04	0.046
	Ratio of all instances In/outside CTL-epitopes				Ratio of all instances In/outside CTL-epitopes				Ratio of all instances In/outside CTL-epitopes				Ratio of all instances In/outside CTL-epitopes			
	0.52				1.31				0.54				1.034			
	Ratio of nonsynonymous to silent mutations In/outside CTL-epitopes				Ratio of nonsynonymous to silent mutations In/outside CTL-epitopes				Ratio of nonsynonymous to silent mutations In/outside CTL-epitopes				Ratio of nonsynonymous to silent mutations In/outside CTL-epitopes			
	1.73				4.65				3				4.65			
	Ratio of Nonsynonymous mutations In/outside CTL-epitopes		Ratio of silent mutations In/outside CTL-epitopes		Ratio of Nonsynonymous mutations In/outside CTL-epitopes		Ratio of silent mutations In/outside CTL-epitopes		Ratio of Nonsynonymous mutations In/outside CTL-epitopes		Ratio of silent mutations In/outside CTL-epitopes		Ratio of Nonsynonymous mutations In/outside CTL-epitopes		Ratio of silent mutations In/outside CTL-epitopes	
	0.66		0.38		2.6		0.57		0.82		0.26		1.77		0.38	
GGG	motif				motif				motif				motif			
	14				14				14				14			
	Normalized Motif Inside CTL-epitopes		Normalized Motif Outside CTL-epitopes		Normalized Motif Inside CTL-epitopes		Normalized Motif Outside CTL-epitopes		Normalized Motif Inside CTL-epitopes		Normalized Motif Outside CTL-epitopes		Normalized Motif Inside CTL-epitopes		Normalized Motif Outside CTL-epitopes	
	0.0058		0.03		0.035		0.015		0.014		0.027		0.023		0.022	
	Motif In/out CTL-epitopes				Motif In/out CTL-epitopes				Motif In/out CTL-epitopes				Motif In/out CTL-epitopes			
	0.19				2.3				0.518				1.045			
	Normalized Inside CTL-epitopes		Normalized Outside CTL-epitopes		Normalized Inside CTL-epitopes		Normalized Outside CTL-epitopes		Normalized Inside CTL-epitopes		Normalized Outside CTL-epitopes		Normalized Inside CTL-epitopes		Normalized Outside CTL-epitopes	
	Nonsynonymous mutations (including stop codon)	Silent mutations	Nonsynonymous mutations (including stop codon)	Silent mutations	Nonsynonymous mutations (including stop codon)	Silent mutations	Nonsynonymous mutations (including stop codon)	Silent mutations	Nonsynonymous mutations (including stop codon)	Silent mutations	Nonsynonymous mutations (including stop codon)	Silent mutations	Nonsynonymous mutations (including stop codon)	Silent mutations	Nonsynonymous mutations (including stop codon)	Silent mutations
	0.035	0.0175	0.053	0.02	0.053	0.013	0.053	0.0152	0.056	0.014	0.051	0.022	0.125	0.0178	0.026	0.013
	Ratio of all instances In/outside CTL-epitopes				Ratio of all instances In/outside CTL-epitopes				Ratio of all instances In/outside CTL-epitopes				Ratio of all instances In/outside CTL-epitopes			
	0.7				0.97				0.75				3.55			
	Ratio of nonsynonymous to silent mutations In/outside CTL-epitopes				Ratio of nonsynonymous to silent mutations In/outside CTL-epitopes				Ratio of nonsynonymous to silent mutations In/outside CTL-epitopes				Ratio of nonsynonymous to silent mutations In/outside CTL-epitopes			
	0.75				1.21				1.73				3.5			
	Ratio of Nonsynonymous mutations In/outside CTL-epitopes		Ratio of silent mutations In/outside CTL-epitopes		Ratio of Nonsynonymous mutations In/outside CTL-epitopes		Ratio of silent mutations In/outside CTL-epitopes		Ratio of Nonsynonymous mutations In/outside CTL-epitopes		Ratio of silent mutations In/outside CTL-epitopes		Ratio of Nonsynonymous mutations In/outside CTL-epitopes		Ratio of silent mutations In/outside CTL-epitopes	
	0.66		0.875		1		0.85		1.09		0		4.8		1.3	
GGT	motif				motif				motif				motif			
	9				9				9				9			
	Normalized Motif Inside CTL-epitopes		Normalized Motif Outside CTL-epitopes		Normalized Motif Inside CTL-epitopes		Normalized Motif Outside CTL-epitopes		Normalized Motif Inside CTL-epitopes		Normalized Motif Outside CTL-epitopes		Normalized Motif Inside CTL-epitopes		Normalized Motif Outside CTL-epitopes	
	0.0058		0.018		0.013		0.015		0.023		0.01		0.0178		0.013	
	Motif In/out CTL-epitopes				Motif In/out CTL-epitopes				Motif In/out CTL-epitopes				Motif In/out CTL-epitopes			
	0.32				0.85				2.23				1.36			
	Normalized Inside CTL-epitopes		Normalized Outside CTL-epitopes		Normalized Inside CTL-epitopes		Normalized Outside CTL-epitopes		Normalized Inside CTL-epitopes		Normalized Outside CTL-epitopes		Normalized Inside CTL-epitopes		Normalized Outside CTL-epitopes	
	Normalized Inside CTL-epitopes	Normalized Outside CTL-epitopes	Normalized Inside CTL-epitopes	Normalized Outside CTL-epitopes	Normalized Inside CTL-epitopes	Normalized Outside CTL-epitopes	Normalized Inside CTL-epitopes	Normalized Outside CTL-epitopes	Normalized Inside CTL-epitopes	Normalized Outside CTL-epitopes	Normalized Inside CTL-epitopes	Normalized Outside CTL-epitopes	Normalized Inside CTL-epitopes	Normalized Outside CTL-epitopes	Normalized Inside CTL-epitopes	no aa changes
	0	0.0175	0.033	0.020	0.026	0.013	0.023	0.023	0.042	0.028	0.0148	0.0148	0.0178	0.035	0.026	0.013

	Ratio of all instances In/outside CTL-epitopes				Ratio of all instances In/outside CTL-epitopes				Ratio of all instances In/outside CTL-epitopes				Ratio of all instances In/outside CTL-epitopes			
	0.33				0.87				2.41				1.32			
	Ratio of nonsynonymous to silent mutations In/outside CTL-epitopes				Ratio of nonsynonymous to silent mutations In/outside CTL-epitopes				Ratio of nonsynonymous to silent mutations In/outside CTL-epitopes				Ratio of nonsynonymous to silent mutations In/outside CTL-epitopes			
	0				2				1.5				0.25			
	Ratio of Nonsynonymous mutations In/outside CTL-epitopes		Ratio of silent mutations In/outside CTL-epitopes		Ratio of Nonsynonymous mutations In/outside CTL-epitopes		Ratio of silent mutations In/outside CTL-epitopes		Ratio of Nonsynonymous mutations In/outside CTL-epitopes		Ratio of silent mutations In/outside CTL-epitopes		Ratio of Nonsynonymous mutations In/outside CTL-epitopes		Ratio of silent mutations In/outside CTL-epitopes	
	0		0.875		1.13		0.56		2.8		1.9		0.68		2.7	
Env																
Motif / HLA	**HLA-A3:01**				**HLA-A2:01**				**HLA-B57:01**				**HLA-B35:01**			
GGA	motif				motif				motif				motif			
	72				72				72				72			
	Normalized Motif Inside CTL-epitopes		Normalized Motif Outside CTL-epitopes		Normalized Motif Inside CTL-epitopes		Normalized Motif Outside CTL-epitopes		Normalized Motif Inside CTL-epitopes		Normalized Motif Outside CTL-epitopes		Normalized Motif Inside CTL-epitopes		Normalized Motif Outside CTL-epitopes	
	0.037		0.026		0.032		0.026		0.033		0.026		0.032		0.027	
	Motif In/out CTL-epitopes 1.42				Motif In/out CTL-epitopes 1.23				Motif In/out CTL-epitopes 1.27				Motif In/out CTL-epitopes 1.14			
	Normalized Inside CTL-epitopes		Normalized Outside CTL-epitopes		Normalized Inside CTL-epitopes		Normalized Outside CTL-epitopes		Normalized Inside CTL-epitopes		Normalized Outside CTL-epitopes		Normalized Inside CTL-epitopes		Normalized Outside CTL-epitopes	
	Nonsynonymous mutations (including stop codon)	Silent mutations	Nonsynonymous mutations (including stop codon)	Silent mutations	Nonsynonymous mutations (including stop codon)	Silent mutations	Nonsynonymous mutations (including stop codon)	Silent mutations	Nonsynonymous mutations (including stop codon)	Silent mutations	Nonsynonymous mutations (including stop codon)	Silent mutations	Nonsynonymous mutations (including stop codon)	Silent mutations	Nonsynonymous mutations (including stop codon)	Silent mutations
	0.070	0.042	0.06	0.011	0.070	0.027	0.066	0.013	0.082	0.011	0.063	0.017	0.068	0.013	0.067	0.0167
	Ratio of all instances In/outside CTL-epitopes				Ratio of all instances In/outside CTL-epitopes				Ratio of all instances In/outside CTL-epitopes				Ratio of all instances In/outside CTL-epitopes			
	1.41				1.21				1.148				0.97			
	Ratio of nonsynonymous to silent mutations In/outside CTL-epitopes				Ratio of nonsynonymous to silent mutations In/outside CTL-epitopes				Ratio of nonsynonymous to silent mutations In/outside CTL-epitopes				Ratio of nonsynonymous to silent mutations In/outside CTL-epitopes			
	0.3				0.51				2				1.3			
	Ratio of Nonsynonymous mutations In/outside CTL-epitopes		Ratio of silent mutations In/outside CTL-epitopes		Ratio of Nonsynonymous mutations In/outside CTL-epitopes		Ratio of silent mutations In/outside CTL-epitopes		Ratio of Nonsynonymous mutations In/outside CTL-epitopes		Ratio of silent mutations In/outside CTL-epitopes		Ratio of Nonsynonymous mutations In/outside CTL-epitopes		Ratio of silent mutations In/outside CTL-epitopes	
	1.16		3.8		1.06		2.07		1.28		0.65		1.01		0.78	
GGG	motif				motif				motif				motif			
	46				46				46				46			
	Normalized Motif Inside CTL-epitopes		Normalized Motif Outside CTL-epitopes		Normalized Motif Inside CTL-epitopes		Normalized Motif Outside CTL-epitopes		Normalized Motif Inside CTL-epitopes		Normalized Motif Outside CTL-epitopes		Normalized Motif Inside CTL-epitopes		Normalized Motif Outside CTL-epitopes	
	0.018		0.017		0.028		0.0148		0.027		0.015		0.009		0.018	
	Motif In/out CTL-epitopes 1.05				Motif In/out CTL-epitopes 1.89				Motif In/out CTL-epitopes 1.8				Motif In/out CTL-epitopes 0.5			
	Normalized Inside CTL-epitopes		Normalized Outside CTL-epitopes		Normalized Inside CTL-epitopes		Normalized Outside CTL-epitopes		Normalized Inside CTL-epitopes		Normalized Outside CTL-epitopes		Normalized Inside CTL-epitopes		Normalized Outside CTL-epitopes	
	Nonsynonymous mutations (including stop codon)	Silent mutations	Nonsynonymous mutations (including stop codon)	Silent mutations	Nonsynonymous mutations (including stop codon)	Silent mutations	Nonsynonymous mutations (including stop codon)	Silent mutations	Nonsynonymous mutations (including stop codon)	Silent mutations	Nonsynonymous mutations (including stop codon)	Silent mutations	Nonsynonymous mutations (including stop codon)	Silent mutations	Nonsynonymous mutations (including stop codon)	Silent mutations
	0.05	0.007	0.03	0.0083	0.06	0.016	0.026	0.006	0.044	0.0055	0.032	0.0088	0.041	0.013	0.0344	0.0076
	Ratio of all instances In/outside CTL-epitopes				Ratio of all instances In/outside CTL-epitopes				Ratio of all instances In/outside CTL-epitopes				Ratio of all instances In/outside CTL-epitopes			
	1.6				2.5				1.22				1.28			
	Ratio of nonsynonymous to silent mutations In/outside CTL-epitopes				Ratio of nonsynonymous to silent mutations In/outside CTL-epitopes				Ratio of nonsynonymous to silent mutations In/outside CTL-epitopes				Ratio of nonsynonymous to silent mutations In/outside CTL-epitopes			
	1.9				0.87				2.2				0.7			
	Ratio of Nonsynonymous mutations In/outside CTL-epitopes		Ratio of silent mutations In/outside CTL-epitopes		Ratio of Nonsynonymous mutations In/outside CTL-epitopes		Ratio of silent mutations In/outside CTL-epitopes		Ratio of Nonsynonymous mutations In/outside CTL-epitopes		Ratio of silent mutations In/outside CTL-epitopes		Ratio of Nonsynonymous mutations In/outside CTL-epitopes		Ratio of silent mutations In/outside CTL-epitopes	
	1.66		0.84		2.57		2.6		1.375		0.625		1.191		1.71	
GGT	motif				motif				motif				motif			
	28				28				28				28			
	Normalized Motif Inside CTL-epitopes		Normalized Motif Outside CTL-epitopes		Normalized Motif Inside CTL-epitopes		Normalized Motif Outside CTL-epitopes		Normalized Motif Inside CTL-epitopes		Normalized Motif Outside CTL-epitopes		Normalized Motif Inside CTL-epitopes		Normalized Motif Outside CTL-epitopes	
	0.007		0.011		0.012		0.010		0.016		0.0098		0.0045		0.0114	
	Motif In/out CTL-epitopes 0.63				Motif In/out CTL-epitopes 1.2				Motif In/out CTL-epitopes 1.6				Motif In/out CTL-epitopes 0.4			
	Normalized Inside CTL-epitopes		Normalized Outside CTL-epitopes		Normalized Inside CTL-epitopes		Normalized Outside CTL-epitopes		Normalized Inside CTL-epitopes		Normalized Outside CTL-epitopes		Normalized Inside CTL-epitopes		Normalized Outside CTL-epitopes	
	Normalized Inside CTL-epitopes	Normalized Outside CTL-epitopes	Normalized Inside CTL-epitopes	Normalized Outside CTL-epitopes	Normalized Inside CTL-epitopes	Normalized Outside CTL-epitopes	Normalized Inside CTL-epitopes	Normalized Outside CTL-epitopes	Normalized Inside CTL-epitopes	Normalized Outside CTL-epitopes	Normalized Inside CTL-epitopes	Normalized Outside CTL-epitopes	Normalized Inside CTL-epitopes	Normalized Outside CTL-epitopes	Normalized Inside CTL-epitopes	no aa changes
	0.007	0.014	0.023	0.011	0.0163	0.027	0.022	0.0074	0.022	0.016	0.020	0.010	0	0.013	0.023	0.011
	Ratio of all instances In/outside CTL-epitopes				Ratio of all instances In/outside CTL-epitopes				Ratio of all instances In/outside CTL-epitopes				Ratio of all instances In/outside CTL-epitopes			
	0.6				1.43				1.22				0.38			

	Ratio of nonsynonymous to silent mutations In/outside CTL-epitopes		Ratio of nonsynonymous to silent mutations In/outside CTL-epitopes		Ratio of nonsynonymous to silent mutations In/outside CTL-epitopes		Ratio of nonsynonymous to silent mutations In/outside CTL-epitopes	
	0.25		0.2		0.68		0	
	Ratio of Nonsynonymous mutations In/outside CTL-epitopes	Ratio of silent mutations In/outside CTL-epitopes	Ratio of Nonsynonymous mutations In/outside CTL-epitopes	Ratio of silent mutations In/outside CTL-epitopes	Ratio of Nonsynonymous mutations In/outside CTL-epitopes	Ratio of silent mutations In/outside CTL-epitopes	Ratio of Nonsynonymous mutations In/outside CTL-epitopes	Ratio of silent mutations In/outside CTL-epitopes
	0.3	1.27	0.74	3.64	1.1	1.6	0	1.18

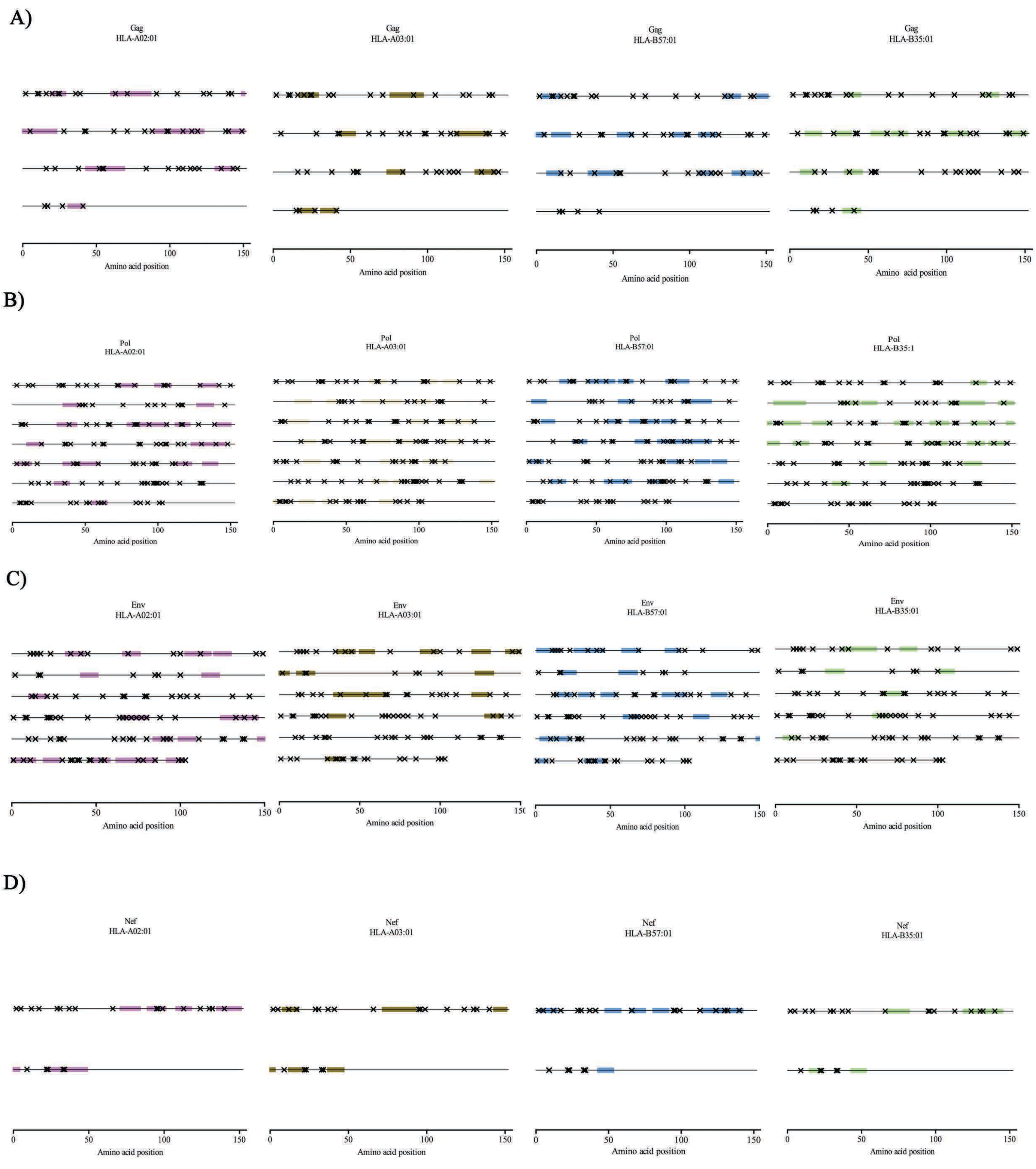
A)
Gag
HLA-A02:01
Gag
HLA-A03:01
Gag
HLA-B57:01
Gag
HLA-B35:01
0
50
100
150
Amino acid position
B)
Pol
HLA-A02:01
Pol
HLA-A03:01
Pol
HLA-B57:01
Pol
HLA-B35:1
C)
Env
HLA-A02:01
Env
HLA-A03:01
Env
HLA-B57:01
Env
HLA-B35:01
D)
Nef
HLA-A02:01
Nef
HLA-A03:01
Nef
HLA-B57:01
Nef
HLA-B35:01

Figure S1

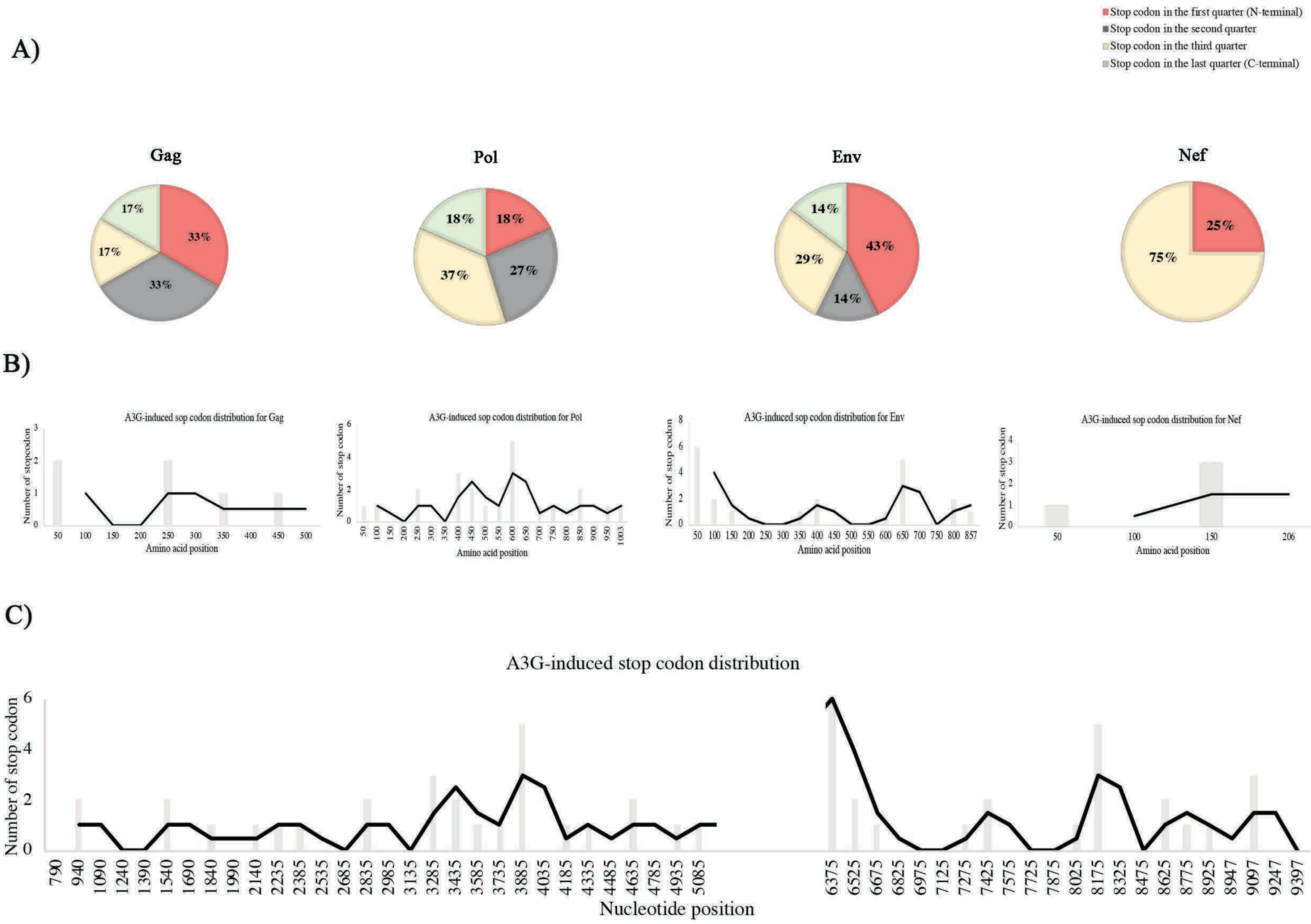
A)
Stop codon in the first quarter (N-terminal)
Stop codon in the second quarter
Stop codon in the third quarter
Stop codon in the last quarter (C-terminal)
Gag
33%
33%
17%
17%
Pol
18%
27%
37%
18%
Env
43%
14%
29%
14%
Nef
25%
75%
B)
A3G-induced sop codon distribution for Gag
Number of stopcodon
Amino acid position
A3G-induced sop codon distribution for Pol
Number of stop codon
Amino acid position
A3G-induced sop codon distribution for Env
Number of stop codon
Amino acid position
A3G-induced sop codon distribution for Nef
Number of stop codon
Amino acid position
C)
A3G-induced stop codon distribution
Number of stop codon
Nucleotide position

Figure S2
